# AI-Driven Predictive Models of Early Recurrence of HCC After Surgical Resection: A Systematic Review

**DOI:** 10.3390/cancers18132028

**Published:** 2026-06-23

**Authors:** Mafalda Mota Neves, Carlos Soares

**Affiliations:** 1 Faculty of Medicine, University of Porto, Alameda Prof. Hernâni Monteiro, 4200-319 Porto, Portugal; carlossoares@med.up.pt; 2 HPB Unit of the General Surgery Department, ULS São João, Alameda Prof. Hernâni Monteiro, 4200-319 Porto, Portugal

**Keywords:** hepatocellular carcinoma, early recurrence, hepatectomy, artificial intelligence, predictive models, PROBAST+AI

## Abstract

**Background/Objectives:** Early recurrence after curative-intent resection is a major determinant of poor prognosis in hepatocellular carcinoma (HCC). Artificial intelligence (AI)-driven predictive models have emerged to identify patients at high risk of recurrence but remain incompletely synthesized for early recurrence specifically. This review aimed to identify and appraise AI-driven models predicting early recurrence after surgical resection. **Methods:** PubMed/MEDLINE, Scopus and Web of Science were searched from inception to November 2025. Eligible studies developed and evaluated AI-driven models predicting early recurrence (≤24 months) after curative-intent hepatectomy as first-line treatment for HCC. Risk of bias and applicability were assessed using PROBAST+AI, and findings were synthesized narratively due to methodological heterogeneity. The review was registered in PROSPERO. **Results:** Thirty-six studies involving 14,716 patients were included. Most studies originated from China (33/36, 91.7%), were single-center (27/36, 75%), and retrospective (35/36, 97.2%). Magnetic resonance imaging (MRI) was the predominant imaging modality (15/36, 41.7%), followed by computed tomography (CT) (11/36, 30.6%) and ultrasound (US)/contrast-enhanced ultrasound (CEUS) (6/36, 16.7%). Three studies developed non-imaging models, and one combined CT and MRI. In within-study comparisons, multimodal models generally showed better discrimination than unimodal approaches. Peritumoral, habitat-based, and multiphasic strategies appeared promising. However, external validation was reported in only 6/36 studies (16.7%), calibration and decision-curve analysis were inconsistently reported, and most studies had high risk of bias. **Conclusions:** AI-driven models show potential to predict early recurrence of HCC after curative-intent resection. Nevertheless, evidence remains limited by methodological heterogeneity and restricted geographical diversity, while clinical utility remains inconsistently evaluated, and no model has yet been generalized in clinical practice. Prospective multicenter studies with standardized outcomes, transparent reporting, and external validation are needed for clinical implementation.

## Simple Summary

Hepatocellular carcinoma often recurs after surgery, especially within the first two years, and early recurrence is usually associated with poorer outcomes. Identifying patients at higher risk may help clinicians plan treatment and follow-up more effectively. In this systematic review, we examined studies that used artificial intelligence to predict early recurrence after liver resection for hepatocellular carcinoma. The available evidence suggests that these models are promising, especially when imaging information is combined with clinical or histopathological data. However, many studies were retrospective, used different methods and outcome definitions, and were rarely validated in independent patient groups. This review highlights both the potential and the current limitations of artificial intelligence in this setting and identifies the main priorities for future research before these tools can be reliably used in clinical practice.

## 1. Introduction

Hepatocellular carcinoma (HCC), which accounts for approximately 75–85% of primary liver cancers, represents a major global health challenge, with liver cancer ranking as the sixth most commonly diagnosed malignancy and the third leading cause of cancer-related mortality worldwide [1,2].

The epidemiology of HCC is characterized by marked geographic variation in underlying etiological factors. Chronic hepatitis B virus infection remains the predominant cause of HCC in East Asia and sub-Saharan Africa, whereas hepatitis C virus infection, alcohol-related liver disease, and metabolic dysfunction-associated steatotic liver disease (MASLD) represent increasingly important etiologies in Western populations [2,3]. These regional differences contribute to significant heterogeneity in tumor biology, disease presentation, and recurrence patterns across patient populations globally [3].

Surgical resection remains a cornerstone of curative-intent treatment for patients with resectable HCC and preserved liver function, particularly when liver transplantation is not considered [2]. However, long-term outcomes remain limited by the high incidence of postoperative tumor recurrence, with recurrence rates approaching 70% within five years after hepatectomy [2,4]. Notably, recurrence remains the principal determinant of long-term survival after resection [2,5]. Accurate prediction of recurrence risk is therefore critical for personalized clinical management, namely by optimizing postoperative surveillance strategies and guiding neoadjuvant or adjuvant treatment decisions.

Tumor recurrence following resection can be categorized into early and late recurrence, which are believed to arise from distinct biological mechanisms [2,4]. Early recurrence is most commonly defined as tumor recurrence within 24 months after surgical resection [2,4], and is thought to result primarily from occult intrahepatic metastases originating from the primary tumor, whereas late recurrence frequently reflects de novo tumorigenesis arising in the chronically diseased liver [2,4,5]. These mechanistic differences have important prognostic implications, as early recurrence is generally associated with more aggressive tumor biology and poorer survival outcomes [4]. Early recurrence occurs in approximately 30–50% of patients undergoing hepatectomy and accounts for up to 70% of postoperative recurrences, reflecting its substantial contribution to treatment failure [4,6].

Several adverse histopathological features have been consistently associated with increased risk of early recurrence following liver resection, including microvascular invasion (MVI), poor tumor differentiation, and the presence of satellite nodules [7]. Among these factors, microvascular invasion has emerged as one of the most important predictors of postoperative recurrence in HCC [7]. However, many of these features can only be definitively determined through postoperative histopathological examination, limiting their usefulness for preoperative risk stratification.

Current clinical decision-making in HCC relies on several treatment guidelines, such as those from the American Association for the Study of Liver Diseases, the European Association for the Study of the Liver (EASL), and the European Society for Medical Oncology (ESMO) [8,9,10], which prioritize a multidisciplinary approach based on staging and prognostic frameworks, including the Barcelona Clinic Liver Cancer (BCLC) classification, as well as liver function assessment tools such as Albumin-Bilirubin (ALBI) and Child-Pugh score [2]. Although these systems provide valuable prognostic information at the population level, and guide treatment allocation, their ability to accurately predict postoperative recurrence risk for individual patients remains limited [11]. This limitation reflects the substantial biological heterogeneity of HCC and the inability of conventional clinicopathological models to capture complex interactions between tumor characteristics, underlying liver disease, and host-related factors [11].

Over the last few years, artificial intelligence (AI)-driven approaches, including machine learning (ML), deep learning (DL), and radiomics, have emerged as promising tools for improving individualized risk prediction in oncology. Deep learning refers to neural network-based methods able to learn feature representations directly from the data [12]. These techniques enable the analysis of high-dimensional clinical, imaging, and histopathological data and may reveal complex patterns associated with tumor behavior that cannot be captured by traditional statistical models [12]. Radiomics models derived from cross-sectional imaging have demonstrated particular promise in HCC by extracting quantitative imaging features that may reflect tumor heterogeneity and microenvironmental characteristics associated with aggressive disease [13].

Reflecting the rapid expansion of this field, several systematic reviews and meta-analyses have evaluated the performance of AI-driven models for predicting recurrence in liver cancer [14,15]. These analyses have reported encouraging predictive performance for early recurrence prediction after surgical treatment, with pooled area-under-the-curve values approaching 0.88 in some studies [14]. Other systematic reviews evaluating artificial intelligence approaches for predicting recurrence after hepatectomy have similarly highlighted the growing role of machine learning models in postoperative risk prediction [16]. Broader reviews examining AI applications across liver cancer have assessed recurrence prediction across multiple treatment modalities, including surgical resection, ablation, and transarterial therapies [15].

Despite these promising findings, several important limitations remain within the literature. First, many reviews combine heterogeneous patient populations including different primary and secondary liver malignancies such as hepatocellular carcinoma, intrahepatic cholangiocarcinoma, and colorectal liver metastases [15]. Second, several analyses include patients treated with different therapeutic modalities despite important biological differences in recurrence mechanisms across treatment strategies [15]. Third, previous reviews frequently evaluate overall recurrence without distinguishing between early and late recurrence, even though these entities arise from distinct biological mechanisms [4,16].

Finally, methodological limitations across primary studies, including retrospective study designs, limited external validation, and inconsistent reporting standards, raise concerns regarding the robustness, reproducibility, and clinical applicability of many prediction models [12,13]. Given the rapid expansion of artificial intelligence-driven prediction models, rigorous methodological evaluation is essential to determine their validity, reproducibility, and potential for clinical translation into decision-making frameworks. Consequently, a comprehensive synthesis specifically focusing on artificial intelligence-driven predictive models for early recurrence following curative-intent hepatectomy as the sole first-line treatment for hepatocellular carcinoma remains lacking.

The primary objective of this systematic review is to systematically identify and synthesise the available evidence on artificial intelligence-driven predictive models developed to estimate the risk of early recurrence after curative-intent surgical resection of hepatocellular carcinoma. This review aims to systematically: (i) describe the characteristics of the included models, including data sources, modeling techniques, and validation strategies; (ii) evaluate their predictive performance; (iii) assess methodological quality and risk of bias; and (iv) explore the clinical applicability of preoperative and postoperative prediction models.

Understanding which models demonstrate robust methodological quality and reproducible predictive performance is essential to facilitate the translation of artificial intelligence-driven risk prediction into clinically meaningful decision support, and to clarify their potential role in future clinical decision-making.

## 2. Materials and Methods

### 2.1. Protocol and Registration

This systematic review was conducted and reported in accordance with the Preferred Reporting Items for Systematic Reviews and Meta-Analyses (PRISMA) 2020 statement [17]. The review protocol was prospectively registered on the International Prospective Register of Systematic Reviews (PROSPERO) under registration number CRD420251267463, to promote transparency. It is available at https://www.crd.york.ac.uk/PROSPERO/view/CRD420251267463 (protocol published on 31 of December 2025, accessed on 28 April 2026). Any clarifications or amendments made after initial registration are reflected in the PROSPERO record.

### 2.2. Search Strategy and Information Sources

A comprehensive electronic search was performed across three databases: PubMed/MEDLINE, Scopus, and Web of Science. The search was conducted between 9 and 16 of November 2025, covering each database from inception to search date. PubMed/MEDLINE was accessed via the National Library of Medicine interface, Scopus via Elsevier, and Web of Science Core Collection via Clarivate Analytics. No language or publication year restrictions were applied at the search stage.

The search strategy combined controlled vocabulary (Medical Subject Headings [MeSH] in PubMed) and free-text terms structured around four core conceptual domains: (i) hepatocellular carcinoma and hepatic resection; (ii) artificial intelligence, machine learning, deep learning, and radiomics; (iii) early recurrence; (iv) prediction. Boolean operators (AND, OR) were used to combine search terms within and across domains. The search query was adapted for each database according to its specific syntax and indexing system. The full electronic search strategy for each database is provided in Appendix A (Table A1). No additional search methods were applied.

### 2.3. Eligibility Criteria

Eligibility criteria were predefined according to the Population, Intervention, Comparator, Outcome, and Study design framework adapted for prediction model research and were applied consistently throughout the review process.

**Inclusion criteria**—Studies were included if they met all of the following criteria:

(i) Population: adult patients (≥18 years) with confirmed hepatocellular carcinoma (HCC) undergoing curative-intent hepatectomy as the sole first-line treatment. When the minimum age was not explicitly reported, eligibility was inferred from the reported age distribution. Studies were considered to represent adult populations if the reported mean or median age and corresponding dispersion measures were consistent with adult HCC cohorts and no pediatric population was explicitly indicated.
(ii) The study developed and validated an artificial intelligence (AI)-driven predictive model, including machine learning (ML), deep learning (DL), or radiomics approaches. Radiomics studies were included when ML or DL methods were used for feature selection and/or as the core modeling approach. Non-imaging models, based solely on clinical, laboratory, and/or histopathological variables, were eligible because they constitute a distinct, routinely available and accessible class of AI-driven predictors of early recurrence, complementing imaging-based approaches. Furthermore, because such models rely on a limited set of structured variables rather than high-dimensional imaging features, they are inherently less susceptible to overfitting, a recurrent limitation of radiomics and deep-learning-based models.
(iii) Predictor data: clinical, histopathological and/or imaging (computed tomography [CT], magnetic resonance imaging [MRI], contrast-enhanced ultrasound [CEUS], or other) data applied preoperatively and/or postoperatively.
(iv) Outcome: early recurrence (intrahepatic and/or extrahepatic) was explicitly defined or modelled within 24 months post-resection. Studies defining early recurrence within a shorter timeframe (e.g., ≤12 months) were considered eligible provided the definition was clearly stated and conceptually aligned with early postoperative recurrence. Time-to-event models (e.g., recurrence-free survival) were eligible if recurrence within the first 24 months after surgery was defined as the primary prediction target.
(v) At least one model performance metric was reported (e.g., area under the receiver operating characteristic curve [AUC], concordance index (C-index), accuracy, sensitivity, or specificity).
(vi) Study type: original research studies reporting the development and validation of artificial intelligence-driven prediction models.
(vii) Only published studies were eligible for inclusion.

**Exclusion criteria**—Studies were excluded if any of the following applied:

(i) Patients received any form of preoperative/neoadjuvant or postoperative/adjuvant anticancer therapy (e.g., transarterial chemoembolization, ablation, systemic chemotherapy, targeted therapy, immunotherapy), due to potential confounding effects on recurrence patterns.
(ii) The study population included mixed or secondary hepatic malignancies (e.g., combined hepatocellular-cholangiocarcinoma, intrahepatic cholangiocarcinoma, or hepatic metastases).
(iii) Studies explicitly including pediatric patients were excluded, as pediatric hepatocellular carcinoma differs substantially from adult disease in terms of underlying etiology, tumor biology, and recurrence patterns, potentially limiting the applicability of prediction models developed for adult populations [18].
(iv) The predictive model was exclusively based on traditional statistical methods (e.g., Cox proportional hazards regression or logistic regression), as this review specifically focused on artificial intelligence-driven approaches. Regression methods (e.g., logistic or Cox regression) were considered part of an AI-driven/radiomics pipeline when applied to ML or combined with ML-based feature selection (e.g., LASSO). Models applying these methods solely to conventional clinical variables, without any ML component, were classified as traditional and excluded.
(v) Artificial intelligence was applied exclusively for diagnostic purposes, image segmentation, microvascular invasion prediction, or outcomes other than early recurrence.
(vi) Studies were excluded if early recurrence within 24 months was not defined a priori as the primary prediction target, including those in which it was assessed only through post hoc analyses of overall survival or recurrence-free survival models.
(vii) Study type: literature reviews, systematic reviews, meta-analyses, conference abstracts, editorials, commentaries, letters, case reports, animal studies, or unpublished studies.
(viii) The full text was not available in a language accessible to the review team.
(ix) Retracted publications were excluded if identified at any stage of the review process.

### 2.4. Study Selection

All records retrieved from the database search were imported into Rayyan [19], a web-based platform for systematic review management. The free online version of Rayyan was used to identify and remove duplicate records, before initiating study selection. Study selection was conducted independently by two reviewers in two sequential phases: title and abstract screening, followed by full-text assessment using predefined eligibility criteria. To minimize bias, reviewers were blinded to each other’s decisions until completion of each phase, using Rayyan’s “Blind mode”. Disagreements between reviewers were resolved through discussion until consensus was reached. Reasons for exclusion at each stage were documented. Inter-reviewer agreement during title and abstract screening was quantified using Cohen’s kappa coefficient, and the strength of agreement was interpreted according to Landis and Koch [20].

### 2.5. Data Extraction

A standardized data extraction form was created, pilot-tested on five randomly selected included studies and refined as necessary. Data extraction was independently performed by two reviewers using the standardized form. Discrepancies were resolved through discussion and consensus. Data extraction was completed prior to risk of bias assessment.

The extraction of information was organized into three categories, consistent with the structure of the main summary tables and appendices in the results:

(i) Study characteristics: study identifier (first author(s) and year of publication), country, number of study centers, study design (retrospective or prospective), total sample size, number of early recurrence events, definition of early recurrence, and type of recurrence assessed (intrahepatic and/or extrahepatic).
(ii) Population characteristics: age, sex distribution, predominant etiology of HCC, Child-Pugh classification, and Barcelona Clinic Liver Cancer (BCLC) stage. Additional contextual variables, including key eligibility restrictions, type of hepatectomy, and R0/margin status, were also extracted when reported.
(iii) Prediction model characteristics and indexed model performance (specified at the end of this section): type of data (clinical, histopathological, imaging), imaging modality (CT, MRI, CEUS, or other), predictor timing (preoperative and/or postoperative), type of AI (ML, DL, or radiomics) and modeling approach, presence of external validation, outcome type (binary or time-to-event), and performance metrics with precision estimate of the indexed model.
(iv) Additional methodological variables of indexed model: validation method, calibration assessment, decision curve analysis, overfitting mitigation strategies, interpretability strategies, and final retained predictors.

Age was recorded as described in the original studies, prioritising overall cohort summary measures whenever available. If only subgroup or cohort-specific values were disclosed, these were documented accordingly. Sex distribution and the number of early recurrence events were, when necessary, derived from subgroup-level information. BCLC stage and Child-Pugh class were summarized according to the range of stages or classes described in each study, since reporting formats varied across studies.

For indexed model performance, the metric considered most appropriate for outcome type was obtained. AUC was extracted for binary outcomes, whereas the C-index was extracted for time-to-event analyses. Other metrics were obtained when AUC or C-index were not available. Performance measures of the indexed model were collected as reported in the original studies, without recalculation. When sensitivity and specificity were presented graphically without numerical values, this was transparently documented. No numerical estimation from graphical data was performed.

Data extraction was based exclusively on information reported in the published articles to ensure transparency and reproducibility. Study authors were not contacted for additional data, as the review aimed to synthesise and appraise the evidence based on the information publicly available in the original reports, including the transparency and completeness of model reporting.

Follow-up duration was not systematically extracted because its reporting and definition were highly heterogeneous across studies and frequently incomplete. Because all included studies aimed to predict early recurrence within 24 or 12 months after resection, follow-up beyond the defined time horizon was not considered necessary for outcome ascertainment for the purposes of this review.

To avoid within-study multiplicity and ensure independence across studies, each study contributed a single indexed model-outcome pair. The indexed model was selected according to a predefined methodological hierarchy, consistent with model-level appraisal principles: in studies reporting multiple AI-driven predictive models developed within the same cohort, priority was given to models with independent external validation. When several externally validated models were available, the model identified by the authors as the final or primary model was selected. In the absence of external validation, the model with the highest discriminative performance in the internal validation or test dataset was prioritized for primary analysis. Only the pre-specified indexed model-outcome pair from each study was used for primary tabular presentation, risk-of-bias assessment, and cross-study comparison. Alternative models reported within the same study were retained for descriptive within-study comparison of modeling strategies and general trends, but detailed performance extraction was restricted to the prespecified indexed model.

### 2.6. Risk of Bias Assessment

The methodological quality, risk of bias, and applicability of the included prediction model studies were assessed using Prediction Model Risk Of Bias Assessment Tool for Artificial Intelligence (PROBAST+AI) [21], an artificial intelligence-specific extension of the PROBAST framework, the internationally recommended instrument for evaluating studies that develop, validate, or update diagnostic or prognostic prediction models.

Given that traditional risk-of-bias tools may insufficiently capture methodological issues specific to artificial intelligence, PROBAST+AI ensures that AI-specific sources of bias were consistently evaluated across all included models. The PROBAST+AI guidance was consulted using the materials provided by the PROBAST Development Group (available at https://www.probast.org/, accessed on 28 April 2026). Although PROBAST+AI is a recent extension, it does not constitute a separate risk-of-bias tool; rather, it provides AI-specific guidance for applying the established PROBAST framework to machine learning and deep learning-based prediction models.

Risk-of-bias assessment was conducted at the level of the pre-specified indexed model-outcome pair, in accordance with PROBAST+AI guidance for model-level appraisal. Because PROBAST+AI evaluates individual prediction models rather than entire studies, the indexed model selected during data extraction was carried forward for risk-of-bias assessment, ensuring consistency between data extraction and quality appraisal while maintaining statistical independence across studies. As included studies performed both model development and performance evaluation within the same publication, a combination assessment approach was applied, requiring separate domain-level judgments for the development and evaluation components before deriving an overall judgement for each indexed model.

In line with the original PROBAST structure, PROBAST+AI evaluates four domains: participants (data sources and population representativeness), predictors (definition, measurement, and availability at the time of use), outcome (definition and assessment procedures), and analysis (sample size, model development, validation, and potential overfitting or data leakage).

Each domain was rated as low, high, or unclear risk of bias and concerns regarding applicability. An overall risk-of-bias judgement was assigned to each study: a model was rated as overall high risk of bias if any single domain was judged high risk. Two reviewers independently performed all assessments, and disagreements were resolved through discussion. Risk-of-bias findings were incorporated into the qualitative synthesis to contextualize reported performance metrics and highlight methodological strengths and limitations in the existing evidence base.

### 2.7. Data Synthesis

Given the anticipated heterogeneity in study design, AI methodologies, input data types, model architectures, outcome definitions, and validation strategies across the included studies, a quantitative meta-analysis of pooled model performance was not performed. Instead, a structured narrative synthesis was conducted to summarise and critically appraise the current evidence on AI-driven prediction models for early recurrence after curative-intent hepatectomy for HCC. Results were summarized descriptively and presented in tables where appropriate.

For the narrative synthesis of predictors, indexed model performance, and within-study comparisons with alternative models, studies were first grouped according to the primary data modality (CT, MRI, US/CEUS, multimodal imaging, or structured non-imaging data) and then presented chronologically within each group, from the earliest to the most recent publication year. The synthesis focused on predictors and reported performance of the indexed model, as well as qualitative within-study comparisons with alternative models described by the study authors.

Assessment of reporting bias and certainty of evidence was not undertaken, in accordance with the prespecified PROSPERO protocol, since this review focused on qualitative synthesis and model-level methodological appraisal rather than meta-analysis of pooled effect estimates.

## 3. Results

### 3.1. Study Selection

**Figure 1:**
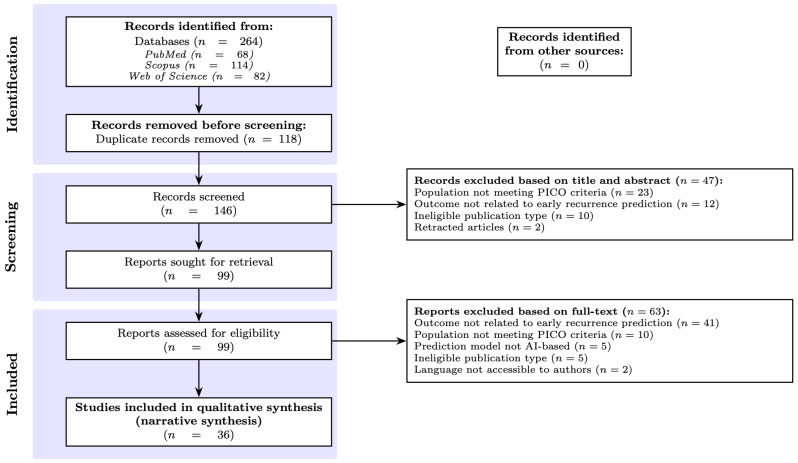
Flow diagram adapted from the PRISMA 2020 statement [17].

Figure 1 depicts the PRISMA flow diagram of the study selection process. Our database search yielded 264 records, including 68 from PubMed, 114 from Scopus, and 82 from Web of Science, with no additional records identified through other sources. After removing 118 duplicates using Rayyan software, 146 records remained for title and abstract screening. At this stage, 47 records were excluded, mainly because the population did not meet the predefined Population, Intervention, Comparator, Outcome, and Study design criteria (*n* = 23), the outcome was not related to early recurrence prediction (*n* = 12), or the publication type was not eligible (*n* = 10). Two retracted articles were also excluded.

A total of 99 reports were assessed for full-text eligibility, and 63 reports were excluded for the following reasons: outcome not related to early recurrence prediction (*n* = 41), population not meeting PICO criteria (*n* = 10), prediction model not AI-driven (*n* = 5), ineligible publication type (*n* = 5), and language not accessible to the review team (*n* = 2). Ultimately, 36 studies were included in the qualitative (narrative) synthesis.

Inter-reviewer agreement during title and abstract screening was almost perfect (Cohen’s κ = 0.89), with 7 disagreements among the 146 screened records (overall agreement: 95.2%). The strength of agreement was interpreted according to Landis and Koch [20].

### 3.2. Study Characteristics

Table 1 summarizes the main characteristics of the included studies. A total of 36 studies comprising 14,716 patients were included in the qualitative synthesis. Most studies were conducted in China (*n* = 33), with one study each originating from Japan [22], Singapore [23], and Taiwan [24]. Publication years ranged from 2018 to 2025, reflecting the recent expansion of AI-driven prediction research in this field.

Most studies were single-center (*n* = 27), whereas nine were multicenter. In terms of study design, almost all were retrospective, with only one prospective study [34]. All studies developed and evaluated AI-driven predictive models for early recurrence after curative-intent resection of hepatocellular carcinoma. Sample sizes varied substantially, from 50 to 4758 patients, indicating considerable heterogeneity in study scale and settings.

Reporting of early recurrence events was inconsistent across studies. Among the studies that provided sufficient information, the total number of early recurrence events, either directly reported or derived from subgroup data, ranged from 20 to 324 cases, corresponding to 19.7% to 60.6% of the study populations.

Definitions of early recurrence also varied across studies. Most studies defined early recurrence using a 24-month postoperative threshold, whereas a smaller number of studies applied a 12-month definition timeframe.

The recurrence type was not uniformly defined across studies. Several studies modelled recurrence without specifying whether intrahepatic or extrahepatic events were included, whereas others explicitly defined the endpoint as including both intrahepatic and extrahepatic recurrence. Four studies reported the distribution of early recurrence events according to intrahepatic or extrahepatic location. In addition, two studies [23,38] focused exclusively on intrahepatic recurrence.

### 3.3. Population Characteristics

Detailed population characteristics of the included studies are summarized in Table 2. Reported age distributions generally fell within the fifth to seventh decades of life. The study cohorts were predominantly male, consistent with the epidemiology of HCC, with male proportions ranging from 73.9% to 92.4%.

The underlying etiology of HCC was predominantly viral across studies. When specified, hepatitis B virus infection was the most common etiology, particularly in Chinese cohorts, whereas hepatitis C virus infection was predominant in the Japanese study [22]. Alcohol-related liver disease and mixed etiologies were also reported, although they were less common.

Patients in the included studies were generally representative of populations undergoing curative-intent hepatectomy for HCC. However, several studies restricted eligibility to narrower clinical, histopathological, or imaging-defined subgroups.

Most patients had preserved liver function (usually corresponding to Child-Pugh class A), and early-stage disease (most frequently corresponding to BCLC 0 or A), although some studies also included selected patients with impaired liver function or more advanced stage tumors. Baseline reporting was, however, incomplete and heterogeneous, and not all studies provided sufficiently detailed information on liver function, stage distribution, or predominant etiology.

Surgical characteristics were less consistently described than demographic and tumor-related variables. Several studies explicitly reported curative resection, radical hepatectomy, or R0 resection, whereas others referred only to hepatectomy or partial hepatectomy without clearly documenting margin status or type of hepatectomy (e.g., anatomic vs. non-anatomic). When reported, the type of hepatectomy ranged from partial resection to more extensive anatomical procedures, including segmentectomy, sectionectomy, and bisectionectomy. Additional details on population eligibility restrictions and surgical characteristics are provided in Appendix A, Table A2.

### 3.4. Prediction Model Characteristics and Performance

A total of 36 studies developed and evaluated artificial intelligence (AI)-driven models for predicting early recurrence of hepatocellular carcinoma after surgical resection. These included 11 CT-based studies, 15 MRI-based studies, 1 study combining CT and MRI, 6 ultrasound (US)- or CEUS-based studies, and 3 non-imaging-based studies. Most models addressed early recurrence as a binary outcome, whereas a smaller number used time-to-event approaches to predict recurrence-free survival within the early recurrence period, modeling recurrence risk over follow-up rather than as a fixed yes/no outcome within a predefined time window. Discriminative performance was most commonly reported using the area under the curve (AUC), whereas survival models more often used the C-index. All studies reported internal validation, but external validation was uncommon. Most models were designed for preoperative prediction, whereas a smaller group incorporated both preoperative and postoperative variables, often through the addition of histopathological findings. This predictor timing defines the intended clinical use case (preoperative decision-making versus postoperative surveillance). Key characteristics and performance metrics of the indexed prediction models are summarized in Table 3 and Table 4, with additional methodological details provided in Appendix A, Table A3.

#### 3.4.1. CT-Based Models

Eleven studies developed prediction models using CT as the primary imaging modality. The earliest study in this group, Wang et al. (2020) [25], reported a deep learning model combining multiphasic CT features with clinical variables. Both modalities underwent simultaneous feature selection, and the final fusion model achieved an AUC of 0.833. The fusion approach showed higher discriminative performance than both the image-only and clinical-only models.

In Lee et al. (2021) [24], preoperative and postoperative models integrating CT-derived radiomic features with clinical variables were developed. The postoperative model achieved a slightly higher AUC (0.741) than the preoperative model after the addition of histopathological features.

In Wu et al. (2022) [26], the best-performing model combined CT-derived radiomic features, Edmondson grade, and tumor size, achieving an AUC of 0.948. This multimodal model had higher discrimination capacity than simpler single-domain versions.

Also published in 2022, Wang et al. (2022) [27] described a deep learning model based on a convolutional neural network (CNN) combining multiphasic CT with clinical data. The model achieved an AUC of 0.869 using a dual attention mechanism across and within imaging phases and was reported to have higher discrimination than both the clinical model and the image-only CNN comparator.

In Cui et al. (2022) [28], a single-phase CT-based three-dimensional CNN approach was used to construct two models with different segmentation techniques. The model based on manual tumor segmentation without inclusion of adjacent liver tissue showed better discriminative performance, with an AUC of 0.789.

In Kinoshita et al. (2023) [22], a deep learning model combining preoperative arterial-phase CT imaging with clinical variables achieved an AUC of 0.710 and showed better discrimination than the corresponding unimodal approaches.

In Kang et al. (2023) [29], radiomics models were developed using six tumor and peritumoral regions of interest (ROI). The ROI comprising the tumor plus 3 mm of the peritumoral tissue was reported to have the highest performance. The final combined model integrated imaging-derived features with alpha-fetoprotein (AFP) and MVI, achieving a validation AUC of 0.830 and yielding higher discrimination than both the imaging-only and clinical-only models.

In Yan et al. (2024) [30], radiomics, clinical, and combined models were developed. After machine learning-based feature selection, the final logistic regression model combined CT-derived features with tumor number, MVI, and the albumin-to-gamma-glutamyl transferase ratio, achieving an AUC of 0.791. This combined model performed better than both the imaging-only and clinical-only models.

Among the most recent studies, Peng et al. (2025) [31] described an externally validated multimodal model based on four-phase CT. Radiomics and deep learning models were first built separately for each CT phase, and then combined into multiphase models. Among the single-phase models, the arterial-phase deep learning model showed the best performance. The final model integrated multiphase radiomic and deep learning features and achieved the best overall performance, with an external validation AUC of 0.930. Adding clinical variables, including AFP and Tumor, Node, Metastasis (TNM) stage, did not further improve model performance.

Also published in 2025, Yao et al. (2025) [32] used a survival modeling framework rather than binary classification. The study developed a purely imaging-based deep learning model using triphasic CT, in which feature extraction and selection were performed end-to-end by the network. Segmentation masks were used to guide the attention mechanism, incorporating the tumor, a 4 mm peritumoral margin, and adjacent liver parenchyma. The model predicted recurrence-free survival, achieving a validation C-index of 0.774 at 24 months.

The second externally validated CT study, Zhang et al. (2025) [33], developed a habitat-based model combining arterial- and portal-phase CT with clinical variables. Tumors were divided into three intratumoral subregions using clustering analysis, and imaging-derived features were extracted separately from each habitat before model construction. The final model integrated multiphase habitat features with age, tumor number, and AFP, and achieved an external validation AUC of 0.896, outperforming both the clinical-only model and the single-phase models.

External validation was reported in only two CT-based studies. Several studies evaluated models combining imaging-derived features with clinical or histopathological variables, often comparing them with unimodal approaches.

#### 3.4.2. MRI-Based Models

Fifteen studies developed prediction models using MRI as the primary imaging modality. The earliest study in this group, Hui et al. (2018) [23], developed a purely imaging-based model using multiparametric preoperative MRI acquired across five phases. Radiomic features were extracted from all phases, but the final model retained a single feature from the T1 equilibrium phase, achieving an overall accuracy of 84%.

In Zhang et al. (2019) [34], a preoperative MRI-based nomogram combined machine learning-selected imaging features with AFP, gross vascular invasion, and non-smooth tumor margin. The final combined model achieved an AUC of 0.841, outperforming both the imaging-only and clinical-radiological models. This study was prospectively designed and used temporal validation.

In Zhao et al. (2021) [35], the best-performing model combined multiparametric MRI-derived features with tumor size, histological grade, and MVI, achieving an internal validation AUC of 0.873.

In Chong et al. (2021) [36], clinical-only, intratumoral, peritumoral, and combined models were compared using multiphase MRI. The peritumoral radiomics model, based on the tumor plus a 1 cm margin, achieved an AUC of 0.842 and showed better discrimination than the intratumoral model. A combined model integrating peritumoral features with clinical, radiological, and histopathological variables showed similar discriminative performance. However, decision curve analysis, which assesses net benefit across threshold probabilities, indicated the highest clinical net benefit for the peritumoral model. The study also compared performance with established prediction systems, including ERASL, TNM stage, and BCLC stage, which were reportedly surpassed by the peritumoral model.

In Li et al. (2022) [37], a preoperative model based on multiparametric MRI was able to achieve an AUC of 0.870. This model combined MRI-derived radiomics features with radiological variables, including tumor-to-portal vein interface, rim enhancement, and tumor capsule. It was reported as having better performance than single-domain comparative models.

In Zhang et al. (2023) [38], a preoperative model based on clinical, laboratory, and MRI-derived radiological variables was developed. Several machine learning algorithms were evaluated using the same set of predictors, and the best-performing model achieved an AUC of 0.706.

Several MRI-based studies were published in 2024. In Wang et al. (2024) [39], a multimodal deep learning model integrating multiparametric MRI with clinical information outperformed both the imaging-only and clinical comparators, achieving an AUC of 0.868. In Wang et al. (2024) [40], an externally validated multimodal model combining MRI with clinical and histopathological variables achieved an AUC of 0.883. In Li et al. (2024) [41], another externally validated model combined MRI-derived features from R2* maps with AFP and MVI, achieving an AUC of 0.827. In Mu et al. (2024) [42], a multi-branch deep learning model based on multiparametric MRI achieved its best performance after tumor size was added, reaching an AUC of 0.842. In Zhao et al. (2024) [43], deep learning outperformed radiomics models, and the highest discrimination was achieved when imaging-derived features were combined with aspartate aminotransferase (AST) and tumor diameter, reaching an AUC of 0.844.

The most recent MRI studies were published in 2025. In Zeng et al. (2025) [44], a multiphase model integrating MRI-derived features with age, AST, MVI, mosaic architecture, and satellite nodules achieved a validation AUC of 0.743 and outperformed single-phased, as well as both the imaging-only and clinical-radiological models. In Wang et al. (2025) [45], a preoperative model incorporating peritumoral features from a tumor plus 5 mm margin together with tumor size and satellite nodules achieved an AUC of 0.850, outperforming intratumoral and imaging-only approaches. In Sun et al. (2025) [46], several machine learning algorithms were compared using clinical and MRI-derived radiological variables, but the conventional statistical model was reported as the best-performing model, with an AUC of 0.785. In Qin et al. (2025) [47], an externally validated hepatobiliary-phase model divided tumors into three intratumoral habitats before feature extraction and combined these with clinical variables, achieving an AUC of 0.820. This model outperformed both the clinical-only model and models that did not incorporate habitat-based analysis.

External validation was reported in three MRI-based studies. Several studies evaluated models combining MRI-derived features with clinical or histopathological variables, and some explored peritumoral or habitat-based feature extraction.

#### 3.4.3. Combined CT- and MRI-Based Model

Only one study used both CT and MRI as imaging inputs. In Wang et al. (2023) [48], a preoperative model integrating multiphasic CT and multiparametric MRI was developed. The CT-only and MRI-only models showed similar performance, whereas the combined CT-MRI model showed higher discrimination. When clinical, radiological, and histopathological variables were added, performance increased further to an AUC of 0.951.

#### 3.4.4. US- and CEUS-Based Models

Six studies used ultrasound or contrast-enhanced ultrasound (CEUS) as the primary imaging modality. The earliest studies in this group were published in 2022. In Zhang et al. (2022) [49], CEUS-based radiomics, deep learning, and combined models were developed to predict 1-year early recurrence. The combined model achieved an AUC of 0.889, and was reported to show higher discrimination than both the deep learning-only and radiomics-only approaches.

In Huang et al. (2022) [50], CEUS-based deep learning features were extracted after manual tumor segmentation and combined with satellite nodules detected on CEUS. Although this appeared to improve performance relative to the unimodal approaches, discrimination remained modest, with a test AUC of 0.568.

In Cao et al. (2024) [51], a clinical-ultrasonic model, an ultrasonic radiomics model, and a combined model were compared. The combined approach achieved the strongest performance, with a validation AUC of 0.925.

In Huang et al. (2024) [52], multiple features were evaluated for their discriminative capacity, including tumor size ≥ 30 mm, satellite nodules on CEUS, and AFP, and were reported to be able to predict early recurrence. The authors also developed an isolated deep learning model, achieving an AUC of 0.547 in internal validation.

The most recent US/CEUS studies were published in 2025. In Liang et al. (2025) [53], a prognostic model integrating Liver Imaging Reporting and Data System (LI-RADS) classification, CEUS findings, and clinicopathological variables achieved C-indices of 0.804 and 0.710 for 1- and 2-year recurrence prediction, respectively. In Liu et al. (2025) [54], phase-specific deep learning models based on CEUS videos were followed by multiphase and combined models. The final model integrating imaging with AFP and albumin showed the best overall performance, with a validation AUC of 0.871, while the multiphase imaging-only model also showed higher discrimination than the single-phase models.

All ultrasound-based studies reported internal validation only. Several models combined CEUS-derived features with clinical variables or deep learning-derived scores.

#### 3.4.5. Non-Imaging-Based Models

Three studies developed prediction models using only clinical, laboratory, and histopathological variables. The earliest study in this group, Mai et al. (2021) [55], developed a model based on clinical and histopathological variables that achieved a validation AUC of 0.736 and outperformed statistical models based on portal hypertension and established recurrence prediction scores, including BCLC and TNM.

In Zeng et al. (2022) [56], a machine learning model based on clinicopathological variables achieved a C-index of 0.762 in internal validation and 0.747 in external validation, and also outperformed the Early Recurrence After Surgery for Liver tumor (ERASL) model, BCLC stage, and TNM stage.

In the most recent study, Zhang et al. (2024) [57], a machine learning model was developed using clinical and histopathological variables with a validation C-index of 0.798, having better reported performance than statistical models based on portal hypertension and ALBI grade.

Only one of the non-imaging-based studies included external validation. Across studies that compared their AI model with conventional prediction approaches (established staging systems and clinical scores such as BCLC, TNM, ALBI or ERASL, and conventional statistical models), the AI-driven model showed superior discrimination in four out of five [36,55,56,57], with one exception [46].

#### 3.4.6. Methodological Reporting

Details regarding validation strategies, calibration assessment, decision curve analysis, interpretability methods, and approaches used to reduce overfitting are summarized in Appendix A, Table A3. Across modalities, most studies prioritized discrimination, whereas reporting of calibration, interpretability, and clinical utility was less consistent.

Calibration, defined as the agreement between predicted and observed outcomes, was assessed in a substantial proportion of studies, most commonly using calibration curves, although reporting was not uniform. Decision curve analysis was performed in several studies to estimate potential clinical net benefit of using the model across different risk thresholds for clinical decision-making.

Reporting of model interpretability also varied. Some deep learning models incorporated visual explanation techniques such as Score-CAM, whereas other studies relied on feature importance measures derived from machine learning models, including approaches such as Shapley Additive Explanations (SHAP). However, several studies did not report any interpretability analysis.

Strategies to mitigate overfitting during model development included feature selection and dimensionality reduction procedures, often using methods such as Least Absolute Shrinkage and Selection Operator (LASSO) regression to limit the number of predictors included in the model. Validation strategies also varied across studies, including split-sample approaches, cross-validation, and other resampling techniques. A consolidated overview of the indexed models by data modality, including discrimination, external validation, and calibration reporting, is provided in Table 5.

### 3.5. Risk of Bias Assessment

**Figure 2:**
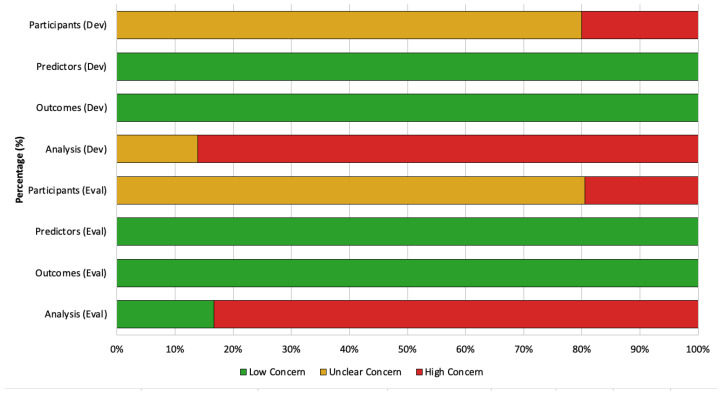
Distribution of Risk of Bias (Percentage per Domain).

Risk of bias and applicability were assessed using the PROBAST+AI combination assessment. Detailed domain-level judgments are presented in Figure 2 and Appendix A, Table A4 and Table A5. Most studies were judged as having high or unclear overall risk of bias, largely driven by concerns in the analysis domain, with additional uncertainty arising from the participants domain. High discrimination alone is insufficient: calibration, decision-curve analysis and clinical net benefit, reported inconsistently here, are equally important for real-world usefulness, so the reported performances should be regarded as preliminary rather than clinically reliable.

Across the included studies, the predictors and outcome domains were consistently judged as low risk of bias in both the development and evaluation components, with predictors clearly defined and outcomes determined in a manner consistent with the review question.

The participants domain was more heterogeneous. Most studies were judged as unclear in both development (Dev) and evaluation (Eval), as cohorts were commonly single-center and regionally specific, resulting in uncertainty regarding representativeness for the review population. High risk of bias in the participants domain was attributed mainly to studies with markedly selected surgical populations, particularly those restricted to AFP-negative HCC, or solitary tumors.

The analysis domain was the main contributor to non-low risk of bias. In model development, this domain was frequently judged as high risk of bias, because of limited sample size relative to model complexity, high-dimensional feature extraction, and insufficient evidence of overfitting control. In a small subset of studies, development analysis was judged as unclear, due to incomplete reporting, particularly regarding missing data handling. In model evaluation, the analysis domain was also frequently rated as high risk of bias, especially when performance relied only on internal split-sample validation, validation cohorts were small, or precision estimates suggested instability of predictive performance. A smaller number of studies were rated as low risk of bias in evaluation analysis when performance was assessed in reasonably sized datasets and discrimination metrics appeared stable.

Applicability concerns arose primarily from the participants domain and were largely driven by the same factors underlying non-low risk-of-bias judgments in that domain. Most studies had at least unclear applicability concerns because they were developed in geographically restricted cohorts, and model transportability may depend on differences in the underlying etiology of HCC across populations. In a small number of studies, unclear applicability concerns were also noted when early recurrence was defined using a 12-month threshold rather than the more commonly used 24-month threshold, not because this definition is inherently inappropriate, but because it is less frequently used and therefore less directly comparable with most studies included in this review. High applicability concern was observed mainly in studies based on markedly selected populations that were narrower than the target population of patients undergoing curative-intent resection.

## 4. Discussion

### 4.1. Overview of Main Findings

This systematic review synthesized current evidence on artificial intelligence-driven models developed specifically to predict early recurrence after curative-intent resection for hepatocellular carcinoma. Overall, the literature suggests that AI-driven prediction is promising, particularly when imaging-derived data is integrated with clinical or histopathological variables [24,25]. Across the included studies, multimodal approaches outperformed unimodal models, supporting the view that early recurrence is shaped by multiple interconnected dimensions of tumor behavior and tumor-host interactions. However, the evidence remains methodologically heterogeneous and not yet sufficiently mature for broad clinical implementation. This is due to the predominance of retrospective single-center studies, limited external validation, and inconsistent reporting of calibration, interpretability, and clinical utility.

### 4.2. Early Recurrence as a Distinct Clinical Target

A notable finding of this review is the limited number of studies that explicitly developed AI-driven models with early recurrence as the predefined prediction target. This reflects both a quantitative limitation and a broader conceptual and methodological gap in the literature. AI research in HCC has often been technically ambitious, but not always equally precise in treating early recurrence as a distinct clinical endpoint. This distinction is relevant because early and late recurrence reflect different biological processes with prognostic implications [4,5,58]. The emphasis on early recurrence therefore requires more than technical optimization; it requires clear biological framing and endpoint-specific model development.

A related issue is the lack of uniformity in the definition of early recurrence itself. Although a 24-month threshold was the most common among the included studies, some authors adopted a 12-month definition [26,27]. In prediction modeling, outcome definition and timing directly affect both comparability and model performance. Because HCC literature still lacks a universally accepted cutoff for early recurrence, this heterogeneity complicates benchmarking across models and limits cross-study comparison. Greater agreement on endpoint definition would support more consistent model development and evaluation.

### 4.3. Emerging Technical Patterns Across the Literature

Overall, the field appears to be evolving toward greater technical sophistication. Earlier studies primarily relied on clinical-only variables, conventional machine-learning methods, or radiomics, frequently restricted to a single imaging phase or sequence. In contrast, newer studies have increasingly explored multimodal fusion, deep learning, peritumoral feature extraction [36], habitat-based analysis [33], and multiphasic or multiparametric imaging [32]. This progression suggests a shift towards models that better reflect the biological complexity of early recurrence.

Beyond the trend toward better discrimination in multimodal approaches relative to single-domain models, several other relevant patterns emerged from within-study comparisons. Models incorporating peritumoral regions were often reported to perform better than those based solely on intratumoral features [45], suggesting that prognostically relevant information may also be encoded in the surrounding liver tissue. On the other hand, habitat-based approaches appeared promising as a means of capturing heterogeneity within the tumor itself [47]. Additionally, multiphasic or multisequence imaging tended to perform better than single-phase approaches among CT, MRI, and CEUS-based studies [31]. These findings suggest that early recurrence risk is influenced not just by gross tumor morphology, but also by spatial heterogeneity, tumor-host interaction, and contrast-enhancement dynamics.

Several studies suggested that AI-driven models may outperform statistical models [55] and conventional staging systems and clinical scores [57], likely because they integrate complex imaging and clinical information beyond traditional frameworks. In only one study did a statistical algorithm outperform an AI approach [46]. Quantitatively, among the five studies that benchmarked their AI model against conventional prediction approaches (established staging systems and clinical scores such as BCLC, TNM, ALBI or ERASL, and conventional statistical models), the AI-driven model showed superior discrimination in four [36,55,56,57]. Although improved discrimination alone does not establish clinical utility, these findings indicate potential for future incremental value over conventional prediction approaches.

### 4.4. Clinical Actionability: Preoperative Versus Postoperative Models

The distinction between preoperative and postoperative models deserves particular attention, because it directly affects clinical utility. Models incorporating postoperative or histopathological variables often achieved stronger discrimination than strictly preoperative models, which is unsurprising given the prognostic weight of factors such as microvascular invasion and tumor differentiation [7]. However, these models address a different clinical scenario. Preoperative prediction has the greatest potential to influence patient selection for surgical resection, and treatment allocation (e.g., resection, transplantation, or ablation), operative planning, perioperative strategy, and early consideration of alternative or neoadjuvant approaches. In contrast, postoperative models are more useful for refining surveillance strategies, identifying patients at high risk of early relapse, and potentially selecting candidates for adjuvant treatment. Superior discrimination in postoperative models should therefore not automatically be interpreted as having greater clinical impact. Rather, it highlights a trade-off between predictive performance and preoperative clinical utility. Microvascular invasion illustrates this particularly well, as it is strongly associated with aggressive biology and postoperative recurrence, yet remains primarily a postoperative histopathological finding [7].

The predictors most frequently included as final model features across studies were consistent with current clinical understanding of recurrence risk after HCC resection. Frequently retained predictors included AFP, tumor size and number, satellite nodules, some liver-related biomarkers, and imaging features suggestive of a more aggressive phenotype [56]. Postoperative models often also incorporated microvascular invasion, tumor grade, or other histopathological markers of more adverse tumor biology. This coherence is reassuring, as it suggests that AI models are not operating independently from existing oncological knowledge, but are instead reassembling clinically plausible predictors within more complex modeling frameworks.

### 4.5. Interpretability, Performance Reporting, and Clinical Readiness

Limited interpretability remains a major obstacle to clinical adoption of AI-driven models. Deep learning approaches may capture complex image representations that reflect tumor heterogeneity more effectively than handcrafted radiomics or conventional semantic imaging assessment, but this gain in representational power comes at the cost of reduced transparency. This is particularly problematic in relatively small retrospective datasets, where complex models may learn unstable or dataset-specific patterns rather than robust biological signals. Although some of the included studies attempted to improve transparency, formal interpretability analyses remained inconsistent, and many reports did not clearly specify the final predictors retained in the model.

In general, the literature seems stronger at demonstrating discrimination than at establishing clinical readiness. Good AUCs or C-indices are encouraging, but they are not sufficient on their own to justify implementation. Current methodological guidance for prediction model evaluation emphasises that performance assessment should include calibration and, ideally, some measure of clinical decision utility such as net benefit. Omission of these dimensions weakens judgments about real-world usefulness. In the included studies, calibration and decision curve analysis were reported inconsistently, and several studies showed a marked decline in performance between training and validation datasets, suggesting residual overfitting and limited generalisability.

Notably, despite a rapidly expanding body of literature, no AI-driven model for early recurrence of HCC has yet been translated into routine clinical practice. This gap between abundant model development and absent clinical implementation reflects the recurring methodological limitations identified in this review, namely frequent overfitting, scarce external validation, limited interpretability, and incomplete reproducibility, which together undermine confidence in real-world performance and currently preclude clinical adoption.

### 4.6. Risk of Bias and Applicability of the Evidence Base

Risk of bias and applicability concerns were substantial across the reviewed literature. Most included studies were retrospective, and only a small minority used prospective designs, temporal validation, or external multicenter validation.

The analysis domain was the most frequent source of concern, reflecting limited sample size relative to model complexity, incomplete control of overfitting, and insufficient reporting of modeling decisions and missing data handling. This pattern is consistent with the structure of PROBAST+AI, in which the analysis domain often captures optimism in reported performance.

Applicability was also constrained by the marked predominance of Asian cohorts, particularly from China, in which HBV-related HCC was the dominant disease background. This raises concerns about transportability. Because etiology shapes tumor biology and recurrence patterns, models developed almost exclusively in HBV-related Chinese cohorts may not transfer reliably to populations in which MASLD, alcohol-related liver disease, or HCV are more prevalent [3]. In addition, the surgical context was often insufficiently reported, particularly regarding type of hepatectomy, extent of resection, and resection margin status, all of which may influence recurrence risk and complicate study comparison. These concerns may directly affect whether a model can reasonably be judged as applicable to the intended clinical setting.

### 4.7. Limitations of This Review

This review also has its limitations. First, the marked heterogeneity in endpoint definitions, imaging modalities, modeling strategies, and reported performance metrics precluded formal quantitative synthesis, limiting this review to a qualitative approach. Second, this review deliberately excluded studies involving preoperative anticancer treatment to preserve clinical and methodological homogeneity. However, this also narrows the applicability of the findings to treatment-naive surgical populations. Third, this review depended on the completeness and clarity of reporting in the primary studies, and some potentially relevant methodological details were insufficiently described. In addition, in the absence of external validation the indexed model corresponded to the best-performing model in internal validation, which may bias the synthesis toward optimistic estimates. The external-validation status of each indexed model is reported in Table 3 and Table 4. Finally, the literature search was conducted up to November 2025, in accordance with the prospectively registered protocol. Studies published after this date were therefore not included.

### 4.8. Implications for Future Research and Practice

Altogether, current evidence suggests that the AI-driven predictive field is evolving rapidly, with consistent patterns emerging across studies despite substantial methodological limitations. Rather than pointing to a single superior algorithmic family, the literature suggests that better-performing models are those that integrate complementary sources of information, capture biologically relevant spatial heterogeneity, and are built around clinically meaningful endpoints.

From a clinical perspective, the most valuable future direction is likely the development of robust, transparent, and externally validated preoperative models capable of informing decisions before surgery. Postoperative models will remain clinically relevant, particularly for surveillance stratification and research on adjuvant strategies, but they serve a different purpose from models intended to guide preoperative decision-making. Future work should prioritise harmonized definitions of early recurrence, clearer reporting of final retained predictors and modeling steps, systematic assessment of calibration and net benefit, and validation across geographically and etiologically diverse populations. The systematic incorporation of immune and inflammatory biomarkers reflecting the host immune state at diagnosis, such as the tumor immune microenvironment and circulating inflammatory indices, represents a further promising avenue for future models. Ultimately, the true clinical value of these models will only be established when prospective studies demonstrate that AI-guided risk stratification can improve clinically meaningful outcomes through better treatment selection, postoperative management, or surveillance.

From a methodological standpoint, the current evidence is constrained by the predominance of retrospective designs and heterogeneous, non-standardised imaging protocols. Higher-quality evidence would come from prospective studies using standardised acquisition, such as complete gadoxetic-acid (Gd-EOB-DTPA) MRI protocols, and adequate follow-up. Future models could also exploit hepatobiliary-phase information that conventional, arterial-phase-based criteria do not capture, which may be especially relevant for small, indeterminate nodules. For instance, lesions that remain hypointense on both the portal-venous and hepatobiliary phases despite lacking definite arterial hyperenhancement have been associated with early HCC or high-grade dysplasia [59]. Although usually classified as indeterminate (LR-3/4), they may carry a higher malignant potential. Prospective, multicentre evaluation of such features and their integration into risk-adapted surveillance would be a valuable next step.

## 5. Conclusions

Artificial intelligence-driven predictive models of early recurrence after curative-intent resection of hepatocellular carcinoma show promising performance, particularly when imaging-derived features are combined with clinical or histopathological variables. However, only a small number of studies have been specifically designed with early recurrence as the predefined prediction target, highlighting an important gap in the literature.

Current evidence remains limited by methodological heterogeneity, scarce external validation, inconsistent reporting of calibration and clinical utility, and restricted geographical diversity. Future progress in this field will depend on the development of transparent and externally validated models, particularly in the preoperative setting. Confirming the generalisability of these models across populations with differing underlying liver-disease etiologies will be equally important, given the current predominance of single-region cohorts.

Harmonized endpoint definitions, clearer reporting standards, and prospective validation across diverse populations will be essential before these tools can be meaningfully integrated into clinical practice. Until such evidence is available, the clinical role of AI-driven predictive models of early HCC recurrence should be considered encouraging but not yet established.

## Figures and Tables

**Table 1:** Characteristics of included studies.

Study	Country	Center(s)	Sample Size (N)	ER Definition	ER Events (n/N, %)	Recurrence Type
CT-based studies						
Wang et al. (2020) [25]	China	Single-center	167	≤12 months	65/167 (38.9%)	NR
Lee et al. (2021) [24]	Taiwan	Single-center	517	≤24 months	239/517 (46.2%)	NR
Wu et al. (2022) [26]	China	Single-center	132	≤12 months	64/132 (48.5%)	Intrahepatic and extrahepatic
Wang et al. (2022) [27]	China	Single-center	167	≤12 months	65/167 (38.9%)	NR
Cui et al. (2022) [28]	China	Single-center	220	≤24 months	94/220 (42.7%)	NR
Kinoshita et al. (2023) [22]	Japan	Single-center	543	≤24 months	220/543 (40.5%)	Intrahepatic and extrahepatic
Kang et al. (2023) [29]	China	Single-center	160	≤24 months	97/160 (60.6%)	Intrahepatic and extrahepatic
Yan et al. (2024) [30]	China	Single-center	148	≤24 months	NR	Intrahepatic and extrahepatic
Peng et al. (2025) [31]	China	Multi-center (3)	519	≤24 months	206/519 (39.7%)	Intrahepatic and extrahepatic
Yao et al. (2025) [32]	China	Multi-center (5)	302	≤24 months	NR	NR
Zhang et al. (2025) [33]	China	Multi-center (2)	344	≤24 months	NR	Intrahepatic and extrahepatic
MRI-based studies						
Hui et al. (2018) [23]	Singapore	Single-center	50	≤24 months	20/50 (40.0%)	Intrahepatic
Zhang et al. (2019) [34]	China	Single-center	155	≤12 months	75/155 (48.3%)	Intrahepatic and extrahepatic
Zhao et al. (2021) [35]	China	Single-center	113	≤24 months	58/113 (51.3%)	Intrahepatic and extrahepatic
Chong et al. (2021) [36]	China	Single-center	323	≤24 months	91/323 (28.2%)	Intrahepatic and extrahepatic
Li et al. (2022) [37]	China	Single-center	302	≤24 months	141/302 (46.7%)	Intrahepatic and extrahepatic
Zhang et al. (2023) [38]	China	Single-center	371	≤24 months	219/371 (59.0%)	Intrahepatic
Wang et al. (2024) [39]	China	Single-center	165	≤24 months	96/165 (58.2%)	NR
Wang et al. (2024) [40]	China	Multi-center (2)	216	≤24 months	68/216 (31.5%)	NR
Li et al. (2024) [41]	China	Multi-center (2)	202	≤24 months	64/202 (31.7%)	Intrahepatic and extrahepatic
Mu et al. (2024) [42]	China	Single-center	331	≤24 months	70/331 (21.1%)	NR
Zhao et al. (2024) [43]	China	Single-center	165	≤24 months	96/165 (58.2%)	Intrahepatic and extrahepatic
Zeng et al. (2025) [44]	China	Multi-center (2)	239	≤24 months	90/239 (37.7%)	NR
Wang et al. (2025) [45]	China	Single-center	200	≤24 months	66/200 (33.0%)	Intrahepatic and extrahepatic
Sun et al. (2025) [46]	China	Single-center	311	≤24 months	131/311 (42.1%)	NR
Qin et al. (2025) [47]	China	Multi-center (2)	370	≤24 months	73/370 (19.7%)	NR
Combined CT and MRI-based studies						
Wang et al. (2023) [48]	China	Single-center	119	≤24 months	60/119 (50.4%)	Intrahepatic and extrahepatic
US and CEUS-based studies						
Zhang et al. (2022) [49]	China	Single-center	172	≤12 months	68/172 (39.5%)	Intrahepatic and extrahepatic
Huang et al. (2022) [50]	China	Single-center	414	≤12 months	NR	Intrahepatic and extrahepatic
Cao et al. (2024) [51]	China	Single-center	127	≤24 months	27/127 (21.3%)	Intrahepatic and extrahepatic
Huang et al. (2024) [52]	China	Single-center	556	≤24 months	307/556 (55.2%)	Intrahepatic and extrahepatic
Liang et al. (2025) [53]	China	Multi-center (2)	279	≤24 months	NR	Intrahepatic and extrahepatic
Liu et al. (2025) [54]	China	Single-center	115	≤24 months	30/115 (26.1%)	Intrahepatic and extrahepatic
Non-Imaging-based studies						
Mai et al. (2021) [55]	China	Single-center	903	≤24 months	324/903 (35.9%)	Intrahepatic and extrahepatic
Zeng et al. (2022) [56]	China	Multi-center (2)	4758	≤24 months	2033/4758 (42.7%)	NR
Zhang et al. (2024) [57]	China	Single-center	541	≤24 months	199/541 (36.8%)	Intrahepatic and extrahepatic

Abbreviations: ER, early recurrence; NR, not reported.

**Table 2:** Population Characteristics.

Study	Age (Years) *	Sex (%Male)	Predominant Etiology	Child-Pugh	BCLC Stage
CT-based studies					
Wang et al. (2020) [25]	<60: 61.1%; ≥60: 38.9%	83.8%	HBV	A (Predominant), B	0, A (Predominant), B, C
Lee et al. (2021) [24]	61 ± 13.1	81.2%	HBV	A (Predominant), B	0 (Predominant), A, B, C
Wu et al. (2022) [26]	ER: 56.9 ± 11.5 vs. Non-ER: 60.0 ± 10.0 (train); ER: 56.4 ± 11.2 vs. Non-ER: 58.1 ± 1.4 (val)	92.4%	HBV	A (Predominant), B	0 + A (Predominant), B
Wang et al. (2022) [27]	<60: 102/167; ≥60: 65/167	83.8%	HBV	A (Predominant), B	0, A (Predominant), B, C
Cui et al. (2022) [28]	<60: 78.1% (train), 69.0% (val); ≥60: 21.9% (train), 31.0% (val)	87.7%	HBV	NR	NR
Kinoshita et al. (2023) [22]	Median 71 (range 19–87)	73.9%	HCV	A (Predominant), B	NR
Kang et al. (2023) [29]	<50: 68.1%; ≥50: 31.9%	88.8%	HBV	A (Predominant), B	A, B (Predominant)
Yan et al. (2024) [30]	56.07 ± 10.60 (train); 52.36 ± 12.04 (val)	91.2%	HBV	NR	0 + A (Predominant), B, C
Peng et al. (2025) [31]	≤60: 76.4% (train), 81.4% (val); >60: 23.6% (train), 18.6% (val)	87.1%	NR	A (Predominant), B	NR
Yao et al. (2025) [32]	56.31 ± 11.29	77.8%	HBV	A (Predominant), B	NR
Zhang et al. (2025) [33]	56.19 ± 11.17 (center 1); 57.64 ± 9.19 (center 2)	75.3%	NR	NR	NR
MRI-based studies					
Hui et al. (2018) [23]	Mean 67 (range 53–81)	86.0%	HBV	A (Predominant), B	NR
Zhang et al. (2019) [34]	50.06 ± 11.44 (train); 51.02 ± 11.96 (val)	80.0%	HBV	A (Predominant), B	0, A, B (Predominant), C
Zhao et al. (2021) [35]	58.06 ± 10.99	81.4%	Viral	A (Predominant), B	NR
Chong et al. (2021) [36]	ER: 55.4 ± 11.3 vs. Non-ER: 54.3 ± 10.9	86.7%	HBV	A (Predominant), B	0, A, B
Li et al. (2022) [37]	57.2 ± 9.8	81%	Hepatitis B/C Virus	A (Predominant), B	NR
Zhang et al. (2023) [38]	<60: 61.2%; ≥60: 38.8%	75.5%	HBV	NR	NR
Wang et al. (2024) [39]	Range 31–81	83.6%	NR	NR	NR
Wang et al. (2024) [40]	53.33 ± 13.43	90%	NR	NR	NR
Li et al. (2024) [41]	63 (IQR 52–68)	91.3%	HBV	NR	NR
Mu et al. (2024) [42]	56.04 ± 10.62	75.9%	HBV	NR	NR
Zhao et al. (2024) [43]	ER: 58.4 ± 10.6 vs. Non-ER: 58.4 ± 10.6 (train); ER: 57.8 ± 10.8 vs. Non-ER: 56.8 ± 10.3 (val)	83.6%	Viral	A (Predominant), B	NR
Zeng et al. (2025) [44]	56 (IQR 49–64) (train); 54 (IQR 48–61) (val)	86.2%	HBV	NR	NR
Wang et al. (2025) [45]	58.3 ± 10.9	80.0%	HBV	A (Predominant), B	NR
Sun et al. (2025) [46]	57.1 ± 11.0	81.0%	HBV	A (Predominant), B, C	NR
Qin et al. (2025) [47]	55.1 ± 11.3	90.0%	Viral	NR	NR
Combined CT and MRI-based studies					
Wang et al. (2023) [48]	ER: 60.23 ± 12.18 vs. Non-ER: 62.68 ± 9.00	81.5%	HBV	NR	0 + A (Predominant), B
US and CEUS-based studies					
Zhang et al. (2022) [49]	48.3 ± 13.2 (train); 52.9 ± 13.1 (val)	85.5%	Hepatitis B/C Virus	NR	NR
Huang et al. (2022) [50]	53.0 (IQR 45.0–60.0)	90.6%	HBV	NR	NR
Cao et al. (2024) [51]	60.57 ± 10.16	75.6%	HBV	NR	NR
Huang et al. (2024) [52]	52.2 ± 11.5	90.3%	HBV	NR	NR
Liang et al. (2025) [53]	52 (IQR 44–60)	88.5%	HBV	NR	NR
Liu et al. (2025) [54]	55.4 ± 14.6	80.9%	NR	NR	NR
Non-Imaging-based studies					
Mai et al. (2021) [55]	≤60: 70.1%; >60: 29.9%	85.5%	HBV	A (Predominant), B	NR
Zeng et al. (2022) [56]	52.5 ± 10.5	86.3%	HBV	NR	0 + A (Predominant), B, C
Zhang et al. (2024) [57]	55 (IQR 48–62)	86.0%	HBV	NR	0 + A (Predominant), B, C

Abbreviations: BCLC, Barcelona Clinic Liver Cancer; ER, Early Recurrence; Non-ER, Non-Early Recurrence; HBV, hepatitis B virus; HCV, hepatitis C virus; NR, not reported; train, training; val, validation. * Age(Years) Formats: Mean ± Standard Error; Median (Interquartile Range).

**Table 3:** Prediction model characteristics and performance—Part 1: model design.

Study	Data Type	Imaging Modality	Predictor Timing	Type of AI	Modeling	Ext. Val.	Outcome Type
CT-based studies							
Wang et al. (2020) [25]	Clinical + imaging	CT (multiphase)	Pre-op	DL	ResNet	No	Binary
Lee et al. (2021) [24]	Clinical + histopathology + imaging	CT (multiphase)	Pre-op and Post-op	ML-based RAD	SVM	No	Binary
Wu et al. (2022) [26]	Histopathology + imaging	CT (multiphase)	Pre-op and Post-op	ML-based RAD	LASSO + LR	No	Binary
Wang et al. (2022) [27]	Clinical + imaging	CT (multiphase)	Pre-op	DL	CNN (attention)	No	Binary
Cui et al. (2022) [28]	Imaging	CT (multiphase)	Pre-op	DL	3D-CNN	No	Binary
Kinoshita et al. (2023) [22]	Clinical + imaging	CT (single-phase)	Pre-op	DL	DenseNet121 + MLP	No	Binary (ER)
Kang et al. (2023) [29]	Clinical + histopathological + imaging	CT (multiphase)	Pre-op and Post-op	ML-based RAD	LASSO + LR	No	Binary
Yan et al. (2024) [30]	Clinical + imaging	CT (multiphase)	Pre-op and Post-op	ML-based RAD	LASSO + LR	No	Binary
Peng et al. (2025) [31]	Clinical + Imaging	CT (multiphase)	Pre-op	ML-based RAD + DL	SVM + CNN	Yes	Binary
Yao et al. (2025) [32]	Imaging	CT (multiphase)	Pre-op	DL	3D DenseNet + attention	No	Time-to-event (RFS)
Zhang et al. (2025) [33]	Clinical + imaging	CT (multiphase)	Pre-op	ML-based RAD	SVM	Yes	Binary
MRI-based studies							
Hui et al. (2018) [23]	Imaging	MRI (multiparametric)	Pre-op	ML-based RAD	kNN	No	Binary
Zhang et al. (2019) [34]	Clinical + imaging	MRI (multisequence)	Pre-op	ML-based RAD	LASSO + LR	No	Binary
Zhao et al. (2021) [35]	Clinical + histopathology + imaging	MRI (multiparametric)	Pre-op and Post-op	ML-based RAD	LASSO + LR	No	Binary
Chong et al. (2021) [36]	Imaging	MRI (multiparametric)	Pre-op	ML-based RAD	SVM	No	Binary
Li et al. (2022) [37]	Imaging	MRI (multiparametric)	Pre-op	ML-based RAD	LASSO + Cox	No	Binary
Zhang et al. (2023) [38]	Clinical + imaging	MRI (NR)	Pre-op	ML	XGBoost	No	Binary
Wang et al. (2024) [39]	Imaging	MRI (multiphase)	Pre-op	DL	ResNet + SSL + attention	No	Binary
Wang et al. (2024) [40]	Clinical + histopathology + imaging	MRI (multiphase)	Pre-op and Post-op	DL	Tensor fusion (MMO loss)	Yes	Binary
Li et al. (2024) [41]	Clinical + histopathology + imaging	MRI (uniparametric)	Pre-op and Post-op	ML-based RAD	LASSO + LR	Yes	Binary
Mu et al. (2024) [42]	Clinical + imaging	MRI (multiparametric)	Pre-op	DL	ResNet (8 branches)	No	Binary
Zhao et al. (2024) [43]	Clinical + imaging	MRI (multiphase)	Pre-op	ML-based RAD + DL	LASSO + ResNet	No	Binary
Zeng et al. (2025) [44]	Clinical + Imaging	MRI (multiphase)	Pre-op	ML-based RAD	LASSO + LR	No	Binary
Wang et al. (2025) [45]	Clinical + imaging	MRI (multiphase)	Pre-op	ML-based RAD	CatBoost	No	Binary
Sun et al. (2025) [46]	Clinical + imaging	MRI (NR)	Pre-op	N/A	LR	No	Binary
Qin et al. (2025) [47]	Clinical + imaging	MRI (single-phase)	Pre-op	ML-based RAD	LightGBM	Yes	Binary
Combined CT and MRI-based studies							
Wang et al. (2023) [48]	Clinical + histopathology + imaging	CT + MRI	Pre-op and Post-op	ML-based RAD	SVM (MRMR + LASSO)	No	Binary
US and CEUS-based studies							
Zhang et al. (2022) [49]	Imaging	CEUS (NR)	Pre-op	DL + ML-based RAD	ResNet-50 + LASSO	No	Binary
Huang et al. (2022) [50]	Clinical + imaging	CEUS (single-phase)	Pre-op	DL-based RAD	DL features + LR	No	Binary
Cao et al. (2024) [51]	Clinical + imaging	US + CEUS (single-phase)	Pre-op	ML-based RAD	LASSO + LR	No	Binary (≤2 years)
Huang et al. (2024) [52]	Imaging	CEUS (multiphase)	Pre-op	DL	DL features + Cox regression	No	Binary
Liang et al. (2025) [53]	Clinical + histopathology + imaging	CEUS (single-phase)	Pre-op and Post-op	ML	GBM	No	Time-to-event (RFS)
Liu et al. (2025) [54]	Clinical + Imaging	CEUS (multiphase)	Pre-op	DL	CNN	No	Binary
Non-Imaging-based studies							
Mai et al. (2021) [55]	Clinical + histopathology	N/A	Pre-op and Post-op	ML	ANN/MLP	No	Binary
Zeng et al. (2022) [56]	Clinical + histopathology	N/A	Pre-op and Post-op	ML	Random Survival Forest	Yes	Time-to-event (RFS)
Zhang et al. (2024) [57]	Clinical + histopathology	N/A	Pre-op and Post-op	ML	Random Survival Forest	No	Time-to-event (RFS)

Abbreviations: DL, Deep learning; Ext. Val., External Validation; ML, Machine Learning; N/A, Not Applicable; RAD, Radiomics.

**Table 4:** Prediction model characteristics and performance—Part 2: validation performance.

Study	Val. Performance Metric with Precision *	Sensitivity	Specificity
CT-based studies			
Wang et al. (2020) [25]	Int.: AUC 0.8331 ± 0.03	NR	NR
Lee et al. (2021) [24]	Int.: AUC 0.741 (precision NR)	NE (ROC curve)	NE (ROC curve)
Wu et al. (2022) [26]	Int.: AUC 0.948 (0.830–0.993)	85.0%	85.7%
Wang et al. (2022) [27]	Int.: AUC 0.869 ± 0.03	NR	NR
Cui et al. (2022) [28]	Int.: AUC 0.789 (0.637–0.941)	61.9%	90.5%
Kinoshita et al. (2023) [22]	Int.: AUC 0.73 (precision NR)	NE (ROC curve)	NE (ROC curve)
Kang et al. (2023) [29]	Int.: AUC 0.830 (0.709–0.952)	78.1%	75.0%
Yan et al. (2024) [30]	Int.: AUC 0.791 (0.650–0.932)	NE (ROC curve)	NE (ROC curve)
Peng et al. (2025) [31]	Int.: NR; Ext.: AUC 0.930 (0.876–0.984)	NE (ROC curve)	NE (ROC curve)
Yao et al. (2025) [32]	Int.: C-index 0.774 (precision NR)	NE (ROC curve)	NE (ROC curve)
Zhang et al. (2025) [33]	Int.: AUC 0.817 (0.7335–0.9009); Ext.: AUC 0.896 (0.8108–0.9817)	Int.: 92.5%; Ext.: 89.8%	Int.: 64.6%; Ext.: 83.3%
MRI-based studies			
Hui et al. (2018) [23]	Accuracy 84% (precision NR)	NR	NR
Zhang et al. (2019) [34]	Int.: AUC 0.841 (0.722–0.959)	91.3%	75.0%
Zhao et al. (2021) [35]	Int.: AUC 0.873 (0.756–0.989)	72.2%	88.2%
Chong et al. (2021) [36]	Int.: AUC 0.842 (0.736–0.951)	60.4%	90.9%
Li et al. (2022) [37]	Int.: AUC 0.870 (0.790–0.940)	88.6%	74.5%
Zhang et al. (2023) [38]	Int.: AUC 0.706 (0.585–0.827)	85.7%	54.5%
Wang et al. (2024) [39]	Int.: AUC 0.868 (precision NR)	NE (ROC curve)	NE (ROC curve)
Wang et al. (2024) [40]	Int.: NR; Ext.: AUC 0.883 (0.830–0.936)	0.845 (0.818–0.872) external	0.966 (0.951–0.981) external
Li et al. (2024) [41]	Int.: NR; Ext.: AUC 0.827 (0.701–0.924)	86.4%	88.9%
Mu et al. (2024) [42]	Int.: AUC 0.842 (0.734–0.932)	83.3%	87.3%
Zhao et al. (2024) [43]	Int.: AUC 0.844 (0.702–0.987)	80.0%	84.6%
Zeng et al. (2025) [44]	Int.: AUC 0.743 (0.613–0.872)	66.7%	73.9%
Wang et al. (2025) [45]	Int.: AUC 0.850 (0.728–0.944)	60.0%	90.0%
Sun et al. (2025) [46]	Reader 1 Int.: AUC 0.785 (0.670–0.899); Reader 2 Int.: AUC 0.765 (0.639–0.892)	Reader 1: 0.692 (0.515–0.870); Reader 2: 0.692 (0.515–0.870)	Reader 1: 0.778 (0.642–0.914); Reader 2: 0.806 (0.676–0.935)
Qin et al. (2025) [47]	Int.: NR; Ext.: AUC 0.820 (0.715–0.926)	73.3%	79.7%
Combined CT and MRI-based studies			
Wang et al. (2023) [48]	Int.: AUC 0.951 (0.792–0.961)	99.0%	83.3%
US and CEUS-based studies			
Zhang et al. (2022) [49]	Int.: AUC 0.889 (precision NR)	90.0%	66.7%
Huang et al. (2022) [50]	Int.: AUC 0.572 (0.502–0.649)	62.0%	56.0%
Cao et al. (2024) [51]	Int.: AUC 0.925 (0.808–1.000)	77.8%	100.0%
Huang et al. (2024) [52]	Int.: AUC 0.547 (0.472–0.622)	68.1%	70.7%
Liang et al. (2025) [53]	Int.: C-index 0.759 (precision NR)	NE (ROC curve)	NE (ROC curve)
Liu et al. (2025) [54]	Int.: AUC 0.871 (0.751–0.970)	83.0%	82.5%
Non-Imaging-based studies			
Mai et al. (2021) [55]	Int.: AUC 0.736 (0.668–0.803)	72.0%	68.6%
Zeng et al. (2022) [56]	Int.: C-index 0.762 ± 0.011; Ext.: C-index 0.747 ± 0.016	NE (ROC curve)	NE (ROC curve)
Zhang et al. (2024) [57]	Int.: C-index 0.798 (precision NR)	NR	NR

Abbreviations: AUC, Area Under the Curve; C-Index, Concordance index; Ext., External; Int., Internal; NE, Non-Extractable; NR, Non-Reported; Val., Validation. * Val. Performance Metric with Precision Formats: C-Index/AUC value ± standard error; C-Index/AUC value (CI 95%); Int. value, Ext. value.

**Table 5:** Summary of AI-driven early-recurrence prediction models by data modality (indexed models).

Data Modality	Studies (*n*)	External Validation (n/N)	Indexed-Model Performance	Calibration (n/N)	DCA (n/N)	Clinical Readiness
CT	11	2/11	AUC 0.71–0.95 ^†^	6/11	6/11	Limited
MRI	15	3/15	AUC 0.71–0.88	12/15	11/15	Limited
CT + MRI	1	0/1	AUC 0.95	1/1	1/1	Limited
US/CEUS	6	0/6	AUC 0.55–0.93	4/6	5/6	Limited
Non-imaging	3	1/3	C-index 0.74–0.80	2/3	3/3	Limited
All studies	36	6/36	—	25/36	26/36	Not ready for routine use

^†^ One CT study reported a C-index (0.77) rather than AUC. Calibration and decision-curve analysis (DCA) were reported inconsistently; risk of bias was high or unclear in most studies and external validation was reported in only 6/36 studies, none in Western/non-HBV cohorts. AUC, area under the curve; CEUS, contrast-enhanced ultrasound; CT, computed tomography; DCA, decision-curve analysis; MRI, magnetic resonance imaging; US, ultrasound.

## Data Availability

The original contributions presented in this study are included in the article. Further inquiries can be directed to the corresponding author.

## Abbreviations

The following abbreviations are used in this manuscript:

AFP	Alpha-fetoprotein
AI	Artificial intelligence
ALBI	Albumin-Bilirubin
ALT	Alanine aminotransferase
AST	Aspartate aminotransferase
AUC	Area under the curve
BCLC	Barcelona Clinic Liver Cancer
CAM	Class Activation Mapping
CEUS	Contrast-enhanced ultrasound
C-index	Concordance index
CNN	Convolutional neural network
CT	Computed tomography
CV	Cross-validation
DCA	Decision curve analysis
DL	Deep learning
EASL	European Association for the Study of the Liver
ECOG	Eastern Cooperative Oncology Group
ER	Early recurrence
ERASL	Early Recurrence After Surgery for Liver tumor
ESMO	European Society for Medical Oncology
GBDT	Gradient Boosted Decision Tree
GGT	Gamma-glutamyl transferase
GLCM	Gray-level co-occurrence matrix
GLSZM	Gray-level size zone matrix
Grad-CAM	Gradient-weighted Class Activation Mapping
HBV	Hepatitis B virus
HCC	Hepatocellular carcinoma
HCV	Hepatitis C virus
ICC	Intraclass correlation coefficient
LASSO	Least Absolute Shrinkage and Selection Operator
LI-RADS	Liver Imaging Reporting and Data System
LOOCV	Leave-one-out cross-validation
MASLD	Metabolic dysfunction-associated steatotic liver disease
MeSH	Medical Subject Headings
ML	Machine learning
MLP	Multilayer perceptron
MRI	Magnetic resonance imaging
MRMR	Minimum Redundancy Maximum Relevance
MVI	Microvascular invasion
PRISMA	Preferred Reporting Items for Systematic Reviews and Meta-Analyses
PROBAST+AI	Prediction Model Risk Of Bias Assessment Tool for Artificial Intelligence
PROSPERO	International Prospective Register of Systematic Reviews
RFA	Radiofrequency ablation
ROI	Region of interest
RSF	Random survival forest
Score-CAM	Score-weighted Class Activation Mapping
SHAP	SHapley Additive exPlanations
TACE	Transarterial chemoembolization
TNM	Tumor, Node, Metastasis
US	Ultrasound

## Appendix A

**Table A1:** Full electronic search strategy for each database.

Database	Search Date	Search Strategy
PubMed/MEDLINE	9 November 2025	(“Carcinoma, Hepatocellular”[MeSH] OR “Hepatocellular carcinoma”[tiab] OR “HCC”[tiab]) AND (“Recurrence”[MeSH] OR “early recurrence”[tiab] OR “postoperative recurrence”[tiab] OR “tumor recurrence”[tiab]) AND (“Hepatectomy”[MeSH] OR “surgical resection”[tiab] OR “hepatectomy”[tiab]) AND (“Artificial Intelligence”[MeSH] OR “Machine Learning”[MeSH] OR “Deep Learning”[MeSH] OR “radiomics”[tiab] OR “artificial intelligence”[tiab] OR “machine learning”[tiab] OR “deep learning”[tiab]) AND (predict *[tiab] OR “predictive model *”[tiab] OR prognos *[tiab] OR “risk model *”[tiab] OR “prognostic model *”[tiab])
Scopus	9 November 2025	TITLE-ABS-KEY((“hepatocellular carcinoma” OR HCC) AND (“early recurrence” OR “postoperative recurrence” OR “tumor recurrence”) AND (“surgical resection” OR hepatectomy) AND (radiomics OR “artificial intelligence” OR “machine learning” OR “deep learning”) AND (predict * OR “predictive model *” OR prognos * OR “risk model *” OR “prognostic model *”))
Web of Science Core Collection	16 November 2025	TS = ((“hepatocellular carcinoma” OR HCC) AND (“early recurrence” OR “postoperative recurrence” OR “tumor recurrence” OR “tumor recurrence”) AND (“surgical resection” OR hepatectomy) AND (“radiomics” OR “artificial intelligence” OR “machine learning” OR “deep learning”) AND (predict * OR “predictive model *” OR prognos * OR “risk model *” OR “prognostic model *”))

* Truncation symbol used in the database search syntax to retrieve all word endings (e.g., predict * retrieves predict, prediction, predictive, and predicting).

**Table A2:** Key eligibility restrictions, hepatectomy type, and margin status of included studies.

Study	Key Eligibility Restrictions	Hepatectomy Type	R0/Margin Status
CT-based studies			
Wang et al. (2020) [25]	Surgically proven HCC; multiphase CT within 1 month; no preoperative treatment; follow-up ≥ 1 year	Curative partial hepatectomy	Yes
Lee et al. (2021) [24]	Preoperative contrast-enhanced CT required	NR	NR
Wu et al. (2022) [26]	None clearly specified beyond resected HCC	Partial hepatectomy	NR
Wang et al. (2022) [27]	Preoperative contrast-enhanced CT within 1 month; no preoperative HCC treatment; follow-up ≥ 1 year	NR	Yes
Cui et al. (2022) [28]	Solitary HCC only; no macrovascular tumor thrombosis or extrahepatic metastasis	Radical hepatectomy	Yes
Kinoshita et al. (2023) [22]	None beyond curative-intent resection	Partial resection, segmentectomy, sectionectomy, bisectionectomy, trisectionectomy	Yes
Kang et al. (2023) [29]	Enhanced CT within 1 month; no preoperative treatment; tumors near liver border or major vessels excluded	NR	NR
Yan et al. (2024) [30]	AFP-negative HCC only	Radical resection	Yes
Peng et al. (2025) [31]	CEUS-based evaluation required	NR	Yes
Yao et al. (2025) [32]	No prior treatment; complete enhanced CT and follow-up required	Partial hepatectomy	NR
Zhang et al. (2025) [33]	Initial hepatectomy only; postoperative pathology-confirmed HCC; preoperative CT within 1 month; no prior RFA, TACE, or radiotherapy; no distant metastasis	NR	NR
MRI-based studies			
Hui et al. (2018) [23]	Treatment-naïve HCC; preoperative MRI required; tumor size ≥ 1 cm	Hepatectomy/partial hepatectomy	NR
Zhang et al. (2019) [34]	Age > 18 years; primary untreated liver lesion; hepatectomy within 7 days of gadoxetic acid-enhanced MRI; lesion size ≥ 1 cm	NR	NR
Zhao et al. (2021) [35]	None clearly specified beyond resected HCC	Partial hepatectomy	NR
Chong et al. (2021) [36]	No macrovascular invasion, gross bile duct tumor thrombosis, lymph node metastasis, or extrahepatic metastasis; no prior antitumor treatment; adequate MRI within 1 month	Anatomical and non-anatomical	NR
Li et al. (2022) [37]	Good-quality multiparametric MRI required; curative hepatectomy within 1 month after MRI; lesions ≥ 1 cm	NR	NR
Zhang et al. (2023) [38]	Primary HCC only; preoperative contrast-enhanced MRI required; no preoperative antitumor treatment	NR	NR
Wang et al. (2024) [39]	Preoperative multiphase MRI required	NR	NR
Wang et al. (2024) [40]	Preoperative Gd-EOB-DTPA MRI required; no extrahepatic metastasis; no prior liver cancer history	Radical hepatic segmentectomy	Yes
Li et al. (2024) [41]	Single HCC only; preoperative enhanced MRI within 2 weeks; no satellite nodules, multiple tumors, macrovascular invasion, or extrahepatic spread	Partial hepatectomy	NR
Mu et al. (2024) [42]	Solitary HCC ≤ 5 cm only; no vascular invasion or distant metastasis; preoperative MRI within 1 month; no prior HCC treatment	NR	NR
Zhao et al. (2024) [43]	No prior antitumor treatment; preoperative contrast-enhanced MRI within 2 weeks; lesions ≥ 10 mm	NR	Yes
Zeng et al. (2025) [44]	Pathologically confirmed HCC; preoperative Gd-EOB-DTPA MRI within 1 month; no pre-MRI treatment; no macrovascular invasion or extrahepatic metastasis	NR	NR
Wang et al. (2025) [45]	Pathologically confirmed HCC; preoperative contrast-enhanced MRI within 1 month; no prior HCC treatment; follow-up ≥ 2 years	Curative/radical hepatectomy	NR
Sun et al. (2025) [46]	Pathologically confirmed HCC after curative hepatic resection; Gd-EOB-DTPA MRI within 1 month; no prior local-regional treatment	NR	NR
Qin et al. (2025) [47]	Early-stage HCC within Milan criteria only; gadoxetic acid-enhanced MRI required; no prior treatment	Curative hepatectomy	NR
Combined CT and MRI-based studies			
Wang et al. (2023) [48]	Histopathologically confirmed HCC; preoperative contrast-enhanced CT and MRI within 2 weeks; no tumor rupture or macrovascular invasion; follow-up ≥ 2 years	Radical liver segmentectomy	NR
US and CEUS-based studies			
Zhang et al. (2022) [49]	Follow-up ≥ 1 year	NR	NR
Huang et al. (2022) [50]	Milan criteria only; CEUS required; no prior ablation or TACE	NR	NR
Cao et al. (2024) [51]	Initial tumor only; preoperative CEUS and CT required; no satellite lesions on CT; surgery within 2 weeks; no perioperative adjuvant therapy	NR	Yes
Huang et al. (2024) [52]	Treatment-naïve cirrhotic patients only; Child-Pugh A only; CEUS within 2 weeks; multifocal HCC excluded	Anatomical and non-anatomical	Yes
Liang et al. (2025) [53]	Solitary HCC only; no vascular invasion, lymph node metastasis, or distant metastasis; preoperative CEUS within 4 weeks	Anatomical and non-anatomical	Yes
US and CEUS-based studies			
Liu et al. (2025) [54]	Early-stage HCC only; BCLC 0/A only; Child-Pugh A/B; ECOG 0-1	Anatomical (partial hepatectomy; margin ≥ 10 mm)	Yes
Non-Imaging-based studies			
Mai et al. (2021) [55]	Child-Pugh A/B only; no prior locoregional or systemic treatment; no major vascular or adjacent-organ invasion	NR	NR
Zeng et al. (2022) [56]	Child-Pugh A/B7; no extrahepatic metastasis; no preoperative anticancer treatment	Primary hepatectomy	Yes
Zhang et al. (2024) [57]	None clearly specified beyond hepatectomy for HCC	NR	Yes

Abbreviations: AFP, Alpha-fetoprotein; BCLC, Barcelona Clinic Liver Cancer; ECOG, Eastern Cooperative Oncology Group; Gd-EOB-DTPA, Gadoxetic acid; HCC, Hepatocellular carcinoma; NR, not reported; RFA, Radiofrequency ablation; TACE, transarterial chemoembolization.

**Table A3:** Overfitting mitigation, predictors, validation methods, interpretability, calibration, and decision curve analysis of included studies.

Study	Overfitting Mitigation	N (Final Predictors)	Validation Method	Interpretability Strategies	Calibration	DCA
CT-based studies						
Wang et al. (2020) [25]	Transfer learning (ImageNet pre-training)	7 clinical (age, tumor size, portal vein invasion, N/L ratio, TB, AFP, BCLC) + 3-phase CT	10-fold CV	NR	NR	NR
Lee et al. (2021) [24]	Genetic algorithm feature selection	NR	10-fold CV in training; independent hold-out test (30%)	NR	NR	NR
Wu et al. (2022) [26]	ANOVA + correlation filtering + LASSO + GBDT	3 (Rad-score + Edmondson grade + Tumor size)	Hold-out split 7:3	NR	Yes (Cal. curves; H-L test)	Yes
Wang et al. (2022) [27]	Data augmentation + transfer learning (ImageNet)	NR	Random split	NR	NR	NR
Cui et al. (2022) [28]	Data augmentation + dropout	NR	5-fold CV in training; temporal split (2014–2017 vs. 2018)	Grad-CAM	Yes (Cal. curves with bootstrapping)	NR
Kinoshita et al. (2023) [22]	Data augmentation + grid search	7 (CECT imaging + 6 clinical: Sex, Age, ALT, AFP, Child-Pugh, Platelets)	Randomized split-sample (training/validation/testing)	Grad-CAM; Permutation importance	NR	NR
Kang et al. (2023) [29]	ICC filtering; LASSO feature selection	3 (Rad-score + AFP + MVI)	Random split 7:3	NR	Yes (Cal. curves)	Yes
Yan et al. (2024) [30]	LASSO feature selection	4 (tumor number + MVI + AGPR + RadScore)	Random split	NR	Yes (Cal. curves)	Yes
Peng et al. (2025) [31]	5-fold CV + transfer learning	NR	5-fold CV (internal) + independent external validation	Grad-CAM	NR	Yes
Yao et al. (2025) [32]	Dropout; early stopping	NR	Split by center (1:4); 5-fold CV (of merged sample)	CAM	Yes (Cal. curves; Brier score)	Yes
Zhang et al. (2025) [33]	*t*-test filtering; Pearson correlation filtering; LASSO	5 (artery-rad-hab-sign + portal-rad-hab-sign + Age + Number of tumors + AFP)	Random 7:3 split in Center 1 (internal) + independent external validation (Center 2)	SHAP	Yes (Cal. curves)	Yes
MRI-based studies						
Hui et al. (2018) [23]	NR	1 (S(4,0) SumVarc - texture parameter from equilibrium phase)	LOOCV with 1-nearest neighbor classifier	NR	NR	NR
Zhang et al. (2019) [34]	LASSO feature selection (10-fold CV)	3 (Rad-score + AFP + Gross vascular invasion)	Temporal split (training Jun/2015–May/2017; validation Jun/2017–May/2018)	Nomogram	Yes (Cal. curves; H-L test)	Yes
Zhao et al. (2021) [35]	LASSO feature selection + 5-fold CV	4 (Rad-score + MVI + pathological grading + tumor size)	Split training/validation 7:3	NR	Yes (Cal. curves; H-L test)	Yes
Chong et al. (2021) [36]	Correlation filtering (r > 0.8); LASSO feature selection; 5-fold CV	38 (radiomics signatures from 7-sequence MRI)	5-fold CV	NR	Yes (Cal. curves)	Yes
Li et al. (2022) [37]	ICC filtering; LASSO feature selection	9 (5 fusion radiomics + TTPVI + rim enhancement + capsule + tumor capsule)	Split-sample	Nomogram	Yes (Cal. curves)	Yes
Zhang et al. (2023) [38]	NR	8 (blood glucose, platelets, intratumoral arteries, AFP, liver cirrhosis, GLR, age, number of tumors)	Training/test split 8:2 + 5-fold CV in training	SHAP	Yes (Cal. curves; Brier score)	Yes
Wang et al. (2024) [39]	MLP dimensionality reduction	NR	Training/validation/testing split (7:1:2)	Score-CAM	NR	NR
Wang et al. (2024) [40]	Early stopping + weighted random sampling (class imbalance) + 5-fold CV	NR	5-fold CV (internal) + independent external validation	NR	NR	NR
Li et al. (2024) [41]	ICC filtering; LASSO feature selection	3 (Rad-score + MVI + AFP > 400 ng/mL)	Split by institution (internal) + independent external validation (Institution 2) + 5-fold CV in training	Nomogram	Yes (Cal. curves; H-L test)	Yes
Mu et al. (2024) [42]	Data augmentation + dropout + early stopping + L2 weight decay	2 (Tumor size + DL predicted score)	Hold-out split ∼76/24	NR	Yes (Cal. curves)	NR
Zhao et al. (2024) [43]	Early stopping + learning rate decay + data augmentation	NR	Random split 8:2	Score-CAM	Yes (Cal. curves)	Yes
Zeng et al. (2025) [44]	ICC filtering; Spearman correlation filtering; LASSO; stepwise logistic regression	6 (Age + AST + AP enhancement + Mosaic architecture + Satellite tumors + Radscore)	Random split 7:3	Nomogram	Yes (Cal. curves; H-L test)	Yes
Wang et al. (2025) [45]	LASSO feature selection	20 (peritumoral radiomics features—GLCM/GLSZM texture-based)	Hold-out split 70/30	NR	Yes (Cal. curves; Brier score)	Yes
Sun et al. (2025) [46]	NR	7 (Gender, ALBI grade, AFP, Tumor size, Tumor number, Intra-lesional fat, Periductal enhancement)	10-fold CV + stratified 80/20 train/test split	NR	Yes (Cal. plots with bootstrapping)	Yes
Qin et al. (2025) [47]	Mann-Whitney + Spearman + LASSO (10-fold CV)	11 (fusion features: radiomics + habitat + 1 clinical [liver cirrhosis])	External validation by geographic/center split (Center 1 training; Center 2 validation)	SHAP	Yes (Cal. curves/plots)	Yes
Combined CT and MRI-based studies						
Wang et al. (2023) [48]	MRMR; LASSO; ICC filtering; 5-fold CV	3 (Tumor size + CT infiltrative margin + Radscore^CT&MRI^)	Hold-out split 7:3	Nomogram	Yes (Cal. curves)	Yes
US and CEUS-based studies						
Zhang et al. (2022) [49]	Data augmentation; transfer learning (ImageNet); learning rate decay	27 (11 radiomics + 16 deep learning features)	Hold-out split 7:3	NR	Yes (Cal. curves)	Yes
Huang et al. (2022) [50]	Univariate feature selection; L1-regularized logistic regression	8 (3 clinical: Satellite nodules + Age + CA 19-9; 5 DLR features)	Hold-out split	NR	NR	Yes
Cao et al. (2024) [51]	Forward selection logistic regression; bootstrap 1000	10 (3 clinical/ultrasound: GP73 + Liver cirrhosis + Washout phase; 7 radiomics)	Random split 7:3	Nomogram	Yes (Cal. curves)	Yes
Huang et al. (2024) [52]	MRMR + L1-penalized regression (LASSO)	4 (Tumor size ≥ 30 mm + Satellite nodule + DL radiomics score + AFP ≥ 400)	Random split ∼2.3:1	NR	NR	NR
Liang et al. (2025) [53]	Variable-importance-based feature selection; early stopping (50 rounds); 10-fold CV for tuning	5 (MVI + Tumor size + LI-RADS + Tumor necrosis + Arterial phase enhancement)	Training/validation split 7:3	SHAP; Permutation importance	Yes (Cal. curves)	Yes
Liu et al. (2025) [54]	Data augmentation; L1 & L2 regularization; early stopping; depthwise separable convolution	NR	75/40 split	Grad-CAM	Yes (Cal. curves; H-L test)	Yes
Non-Imaging-based studies						
Mai et al. (2021) [55]	Repeated randomized trials (hyperparameter optimization)	8 (Tumor size, Blood loss, HBV-DNA, AFP, GGT, Tumor differentiation, Satellite nodules, MVI)	Random split 3:1	Feature importance ranking	Yes (Cal. plots)	Yes
Zeng et al. (2022) [56]	RSF variable-importance-based selection	15 (Age, Gender, Etiology, Platelet count, Albumin, Total bilirubin, AFP, Tumor size, Tumor number, MVI, Macrovascular invasion, Edmondson-Steiner grade, Tumor capsular, Satellite nodules, Liver cirrhosis)	Temporal split (internal) + independent external validation	RSF variable importance (VIMP)	Yes (Cal. plot)	Yes
Zhang et al. (2024) [57]	LASSO regression	9 (AFP, GPR, BT, MVI, LVI, ESG, LCI, SN, BCLC)	Training/validation split 7:3	SHAP; Variable importance (VIMP)	NR	Yes

Abbreviations: AFP, alpha-fetoprotein; BCLC, Barcelona Clinic Liver Cancer; BT, bilirubin total; Cal., calibration; CV, cross-validation; GPR, gamma-glutamyl transpeptidase to platelet ratio; H-L test, Hosmer-Lemeshow test; LVI, lymphovascular invasion; LCI, liver cirrhosis index; MVI, microvascular invasion; NR, not reported; Score-CAM, Score-weighted Class Activation Mapping; SHAP, SHapley Additive exPlanations; SN, satellite nodules.

**Table A4:** Risk of bias assessment (PROBAST+AI)—Part 1: Development domains.

Study	Study Participants (RoB)	Predictors (RoB)	Outcomes (RoB)	Analysis (RoB)
CT-based studies				
Wang et al. (2020) [25]	Unclear	Low	Low	High
Lee et al. (2021) [24]	Unclear	Low	Low	High
Wu et al. (2022) [26]	Unclear	Low	Low	High
Wang et al. (2022) [27]	Unclear	Low	Low	High
Cui et al. (2022) [28]	High	Low	Low	High
Kinoshita et al. (2023) [22]	High	Low	Low	High
Kang et al. (2023) [29]	Unclear	Low	Low	High
Yan et al. (2024) [30]	High	Low	Low	High
Peng et al. (2025) [31]	Unclear	Low	Low	High
Yao et al. (2025) [32]	Unclear	Low	Low	High
Zhang et al. (2025) [33]	Unclear	Low	Low	High
MRI-based studies				
Hui et al. (2018) [23]	Unclear	Low	Low	High
Zhang et al. (2019) [34]	Unclear	Low	Low	High
Zhao et al. (2021) [35]	Unclear	Low	Low	High
Chong et al. (2021) [36]	Unclear	Low	Low	High
Li et al. (2022) [37]	Unclear	Low	Low	Unclear
Zhang et al. (2023) [38]	Unclear	Low	Low	High
Wang et al. (2024) [39]	Unclear	Low	Low	High
Wang et al. (2024) [40]	Unclear	Low	Low	High
Li et al. (2024) [41]	High	Low	Low	High
Mu et al. (2024) [42]	High	Low	Low	High
Zhao et al. (2024) [43]	Unclear	Low	Low	High
Zeng et al. (2025) [44]	Unclear	Low	Low	High
Wang et al. (2025) [45]	Unclear	Low	Low	High
Sun et al. (2025) [46]	Unclear	Low	Low	High
Qin et al. (2025) [47]	Unclear	Low	Low	High
Combined CT and MRI-based studies				
Wang et al. (2023) [48]	Unclear	Low	Low	High
US and CEUS-based studies				
Zhang et al. (2022) [49]	High	Low	Low	High
Huang et al. (2022) [50]	Unclear	Low	Low	High
Cao et al. (2024) [51]	Unclear	Low	Low	High
Huang et al. (2024) [52]	Unclear	Low	Low	High
Liang et al. (2025) [53]	High	Low	Low	Unclear
Liu et al. (2025) [54]	Unclear	Low	Low	High
Non-Imaging-based studies				
Mai et al. (2021) [55]	Unclear	Low	Low	Unclear
Zeng et al. (2022) [56]	Unclear	Low	Low	Unclear
Zhang et al. (2024) [57]	Unclear	Low	Low	Unclear

Abbreviations: RoB, Risk of Bias. Domain-level risk of bias rated as Low, High, or Unclear per PROBAST+AI.

**Table A5:** Risk of bias assessment (PROBAST+AI)—Part 2: Evaluation domains and overall judgement.

Study	Study Participants (RoB)	Predictors (RoB)	Outcomes (RoB)	Analysis (RoB)	Overall Risk of Bias	Applicability Concern
CT-based studies						
Wang et al. (2020) [25]	Unclear	Low	Low	High	High	Unclear Concern
Lee et al. (2021) [24]	Unclear	Low	Low	Low	High	Unclear Concern
Wu et al. (2022) [26]	Unclear	Low	Low	High	High	Unclear Concern
Wang et al. (2022) [27]	Unclear	Low	Low	High	High	Unclear Concern
Cui et al. (2022) [28]	High	Low	Low	High	High	High Concern
Kinoshita et al. (2023) [22]	High	Low	Low	High	High	High Concern
Kang et al. (2023) [29]	Unclear	Low	Low	High	High	Unclear Concern
Yan et al. (2024) [30]	High	Low	Low	High	High	High Concern
Peng et al. (2025) [31]	Unclear	Low	Low	Low	High	Unclear Concern
Yao et al. (2025) [32]	Unclear	Low	Low	High	High	Unclear Concern
Zhang et al. (2025) [33]	Unclear	Low	Low	Low	High	Unclear Concern
MRI-based studies						
Hui et al. (2018) [23]	Unclear	Low	Low	High	High	Unclear Concern
Zhang et al. (2019) [34]	Unclear	Low	Low	High	High	Unclear Concern
Zhao et al. (2021) [35]	Unclear	Low	Low	High	High	Unclear Concern
Chong et al. (2021) [36]	Unclear	Low	Low	High	High	Unclear Concern
Li et al. (2022) [37]	Unclear	Low	Low	High	High	Unclear Concern
Zhang et al. (2023) [38]	Unclear	Low	Low	High	High	Unclear Concern
Wang et al. (2024) [39]	Unclear	Low	Low	High	High	Unclear Concern
Wang et al. (2024) [40]	Unclear	Low	Low	High	High	Unclear Concern
Li et al. (2024) [41]	High	Low	Low	High	High	High Concern
Mu et al. (2024) [42]	High	Low	Low	High	High	High Concern
Zhao et al. (2024) [43]	Unclear	Low	Low	High	High	Unclear Concern
Zeng et al. (2025) [44]	Unclear	Low	Low	High	High	Unclear Concern
Wang et al. (2025) [45]	Unclear	Low	Low	High	High	Unclear Concern
Sun et al. (2025) [46]	Unclear	Low	Low	High	High	Unclear Concern
Qin et al. (2025) [47]	Unclear	Low	Low	High	High	Unclear Concern
Combined CT and MRI-based studies						
Wang et al. (2023) [48]	Unclear	Low	Low	High	High	Unclear Concern
US and CEUS-based studies						
Zhang et al. (2022) [49]	High	Low	Low	High	High	High Concern
Huang et al. (2022) [50]	Unclear	Low	Low	High	High	Unclear Concern
Cao et al. (2024) [51]	Unclear	Low	Low	High	High	Unclear Concern
Huang et al. (2024) [52]	Unclear	Low	Low	High	High	Unclear Concern
Liang et al. (2025) [53]	High	Low	Low	High	High	High Concern
Liu et al. (2025) [54]	Unclear	Low	Low	High	High	Unclear
Non-Imaging-based studies						
Mai et al. (2021) [55]	Unclear	Low	Low	Low	Unclear	Unclear
Zeng et al. (2022) [56]	Unclear	Low	Low	Low	Unclear	Unclear Concern
Zhang et al. (2024) [57]	Unclear	Low	Low	Low	Unclear	Unclear Concern

Abbreviations: RoB, Risk of Bias. Domain-level risk of bias rated as Low, High, or Unclear per PROBAST+AI.
